# Causes of death among international travellers in Peru, 2017 to 2021

**DOI:** 10.1093/jtm/taad163

**Published:** 2023-12-21

**Authors:** Kasim Allel, Miguel M Cabada, Collen Lau, Deborah Mills, Richard C Franklin, Yan Zhu, Luis Furuya-Kanamori

**Affiliations:** Department of Disease Control, London School of Hygiene and Tropical Medicine, London WC1E 7HT, UK; Institute for Global Health, University College London, London WC1E 6BT, UK; UQ Centre for Clinical Research, Faculty of Medicine, The University of Queensland, Brisbane 4029, Australia; Division of Infectious Diseases, University of Texas Medical Branch, Galveston, TX 77555, USA; Cusco Branch – Alexander von Humboldt Tropical Medicine Institute, Universidad Peruana Cayetano Heredia, Cusco, Peru; UQ Centre for Clinical Research, Faculty of Medicine, The University of Queensland, Brisbane 4029, Australia; Dr Deb The Travel Doctor, Travel Medicine Alliance, Brisbane 4000, Australia; College of Public Health, Medical and Veterinary Sciences, James Cook University, Townsville 4811, Australia; UQ Centre for Clinical Research, Faculty of Medicine, The University of Queensland, Brisbane 4029, Australia; Zhuhai International Travel Healthcare Center of China Customs, Zhuhai 519020, China; UQ Centre for Clinical Research, Faculty of Medicine, The University of Queensland, Brisbane 4029, Australia

**Keywords:** Prevention, America, communicable diseases, non-communicable diseases, injuries

## Abstract

**Background:**

The wellbeing and safety of international tourists is a paramount concern for governments and stakeholders. Mortality among travellers and the causes of death serve as a significant metric of destination safety. We describe the epidemiology and causes of death among international travellers in Peru.

**Methods:**

Data retrieved from the Peruvian government’s deaths certificates registry included all non-residents who died between January 2017 and December 2021. We analysed the national incidence and causes of death among international travellers in Peru. Causes of death were classified into non-communicable diseases (NCD), communicable diseases and injuries. We classified fatalities according to the existence of preventive measures that could be provided during the travel medicine consultation to decrease the risk.

**Results:**

We obtained records from 1514 deaths among international travellers (973 males, 64%). The incidence increased from 0.2 deaths per 10 000 travellers in 2017 to 9.9 in 2021. NCDs were the most common causes of death (*n* = 560, 37%), followed by communicable diseases (*n* = 487, 32%), and injuries (*n* = 321, 21%). Causes of death were unknown in 9.7% of the records. The leading causes of death in these categories were cancer, cardiovascular disease, COVID-19 and trauma. We found similar sex distribution of NCDs in travellers aged >50 years and higher rates of communicable diseases among males across all ages. Injury-associated deaths were significantly higher among males aged 18–29 years (*P* < 0.001) compared with other sex-age groups. We estimated that for 57.7% of deaths risk could have been decreased through pre-travel advice.

**Conclusion:**

Rates of deaths among travellers to Peru increased over time. Most deaths were due to NCDs, followed by communicable diseases and injuries. Pre-travel medical optimization and effective advice focused on age-sex and destination specific risks could reduce risk among travellers. Increased awareness among travel medicine practitioners and improvement of emergency medical response systems in Peru could decrease mortality.

## Introduction

In 2022, there were 960 million international arrivals worldwide representing two-thirds of the arrivals registered before the COVID-19 pandemic.^1^ International travellers face a myriad of health risks abroad that can potentially lead to severe illnesses and occasionally to death.^1–4^ It is estimated that between 30 and 50% of these travellers experience health problems abroad, including injuries and accidents.^5^^,^^6^ Although mortality is generally low among travellers, the ever increasing number of international arrivals makes this a growing problem.^3^ Motor vehicle accidents and injuries due to exposure to unfamiliar environments (e.g. drowning, suffocation, trauma) or violence, and non-communicable diseases (NCDs), such as cardiovascular disease (CVD) are among the top causes of mortality in travellers globally.^4^^,^^7–9^ Emerging or re-emerging pathogens such as COVID-19 or Yellow Fever and the increase in population mobility, such as the large refugee crises, have highlighted the importance of some infectious diseases (IDs) as causes of morbidity and mortality among travellers.^10^^,^^11^ Travel stressors may exacerbate chronic medical conditions, cause acute illness such as pulmonary embolisms, or uncover undiagnosed underlying health conditions.^8^^,^^12^ The absence of robust data on injuries and severe illnesses in travellers, which may culminate in fatalities on a global spectrum, hinder the capacity of travel medicine practitioners to confront the escalating numbers of deaths among travellers. Consequently, the preventative measures and educational resources for individuals opting to partake in travel have not been developed.^13^

The region of Latin America received 113 million international travellers in 2017.^14^ These travellers were exposed to unique health challenges associated with a diverse geography, climate and natural environments. The re-emergence of vector-borne diseases such as dengue and Yellow Fever in tropical areas, inadequate sanitation and biosafety, and limited access to emergency and trauma healthcare services pose challenges to the management of acute and chronic illnesses in the region.^15^^,^^16^ This is especially important in countries with a rapidly growing tourism industry such as Peru, which has become a popular destination among travellers within the region and from high-income countries (HIC) around the globe. More than 4 million international arrivals were registered in Peru in 2017 and the numbers have been recovering after the COVID-19 pandemic despite the recent political instability in the country.^14^ Peru embodies the confluence of diverse environmental and epidemiological risk factors affecting travellers, including varied landscapes, ranging from dense jungles and high-altitude regions to bustling urban centres. The heightened risk of tropical diseases such as malaria and Yellow Fever is a prominent concern in the Amazon basin.^17^ Meanwhile, travellers to high-altitude areas such as Cusco may experience altitude related illnesses that can lead to life-threatening complications without proper preparation and care.^18^^,^^19^ The evolving socio-economic and healthcare landscape in Peru also plays a pivotal role in shaping the health outcomes of travellers, with significant disparities in the quality of healthcare services in the main cities of the coast compared with the rest of the country. These differences potentially affect the management of various medical conditions among travellers and affect the number of preventable deaths.^20^^,^^21^ Therefore, it is crucial to understand the causes and epidemiology of death among travellers in Peru to foster a safe and responsive travel environment and guide prevention policies and strategies.

In this article, we evaluate the main causes of death, their geographic distribution and risk factors among international travellers to Peru between 2017 and 2021. This is an exploratory study that aims to increase the understanding of the distinctive risk factors and health challenges faced by travellers that died in Peru.

## Methods

### Study design and data

We analysed deaths that occurred in Peru among international travellers registered in the Peruvian Ministry of Health. We used the Peruvian Government Transparency Portal to request information captured in death certificates.^22^ Certificates registered (Supplementary Figure A1) between 1 January 2017 and 31 December 2021 from people with a reported nationality other than Peruvian were requested. Information on demographics, causes of death and location of the event was collected. Date of death was categorized into year and southern hemisphere seasons (i.e. summer is December to March).

### Causes of death

We collected information about the immediate cause and three underlying causes of death. Immediate cause of death was reported using the International Classification of Diseases (ICD)-10 codes.^23^ To avoid misclassification of the causes of death due to common errors in death certificates,^24^ two investigators (M.M.C. and L.F.K., both medical doctors trained in Peru) reviewed each case and determined the cause of death independently. Discrepancies in the cause of death determinations were discussed and agreed by consensus. Causes of deaths were categorized into communicable diseases, NCDs, injuries or unknown if no determination of the cause of death was reached. We used the Global Burden of Disease Project classification to categorize the causes of death.^25^ Subcategories of communicable diseases included COVID-19, diarrhoea, HIV-associated, lower respiratory infections, meningitis and other IDs (e.g. dengue, brain abscess). Categories for NCDs included autoimmune diseases, cancer, cirrhosis, diabetes mellitus, digestive diseases, haematological diseases, myocardial infarction, neurological diseases, renal diseases, stroke, other CVDs and other NCDs (e.g. obesity, hypertension). Injuries comprised altitude sickness, drowning, other injuries (e.g. electrocution, fall and other traumas), poisoning, road injury, suffocation and violence.

### Demographic and socioeconomic variables

Sociodemographic data retrieved from death certificates included age, sex, marital status, nationality, region in Peru where death occurred, ethnicity and whether an autopsy was conducted. We used the following age group categories <18, 18–29, 30–39, 40–49, 50–59, 60–69, 70–79 and ≥80 years. We classified individual countries of origin according to their income level using World Bank 2023 categorization for high-income and low-and middle-income (LMIC) countries.^26^ We also collected information on education level (none, and completed or uncompleted primary, secondary and tertiary), although these data were missing for 44%.

### Statistical analyses

Descriptive statistics of the number of observations and relative frequencies across all causes of death, demographic and socioeconomic variables were reported. We carried out a spatial visualization of the reported deaths over each of the 25 regional units within Peru. Maps of the distribution of diseases and categories were computed for all study years, and before the onset of the COVID-19 pandemic, provided in Supplementary Material. Incidence plots over time by cause of death categories, age-group, sex and regions were created. We calculated mortality incidence rate for 10 000 travellers utilizing official numbers on international tourists arriving in Peru.^27^ We used the chi-square (χ^2^) tests to identify differences between causes of death categories. Cases with unknown cause of death (*n* = 148, 9.78% of the total sample) were removed from univariate analyses. We calculated the number of deaths for which risk levels could potentially be reduced through effective pre-travel health consultation. For this, we compared avoidable and non-avoidable deaths as defined by the Organization for Economic Cooperation and Development (OECD)^28^ and travel medicine experts (Supplementary Tables A1–2). All calculations were computed in Stata SE version 17.0 (College Station, TX: StataCorp LLC). We used the Geographic Information System Open-Source Geospatial Foundation Project (QGIS) version 2022 for map creation (http://qgis.org).

## Results

### Overall incidence of death among international travellers in Peru

A total of 1514 death certificates from subjects whose nationality was other than Peruvian were retrieved, most of these were male (*n* = 973, 64.3%) with ethnicity reported as mixed (*n* = 1077, 71.1%) (Supplementary Table A3). Those aged 60–69-years (*n* = 260, 17.2%) and single marital status (*n* = 728, 48.7%) groups comprised the largest proportion of deaths compared with other age groups or marital statuses, respectively.

**Figure 1:**
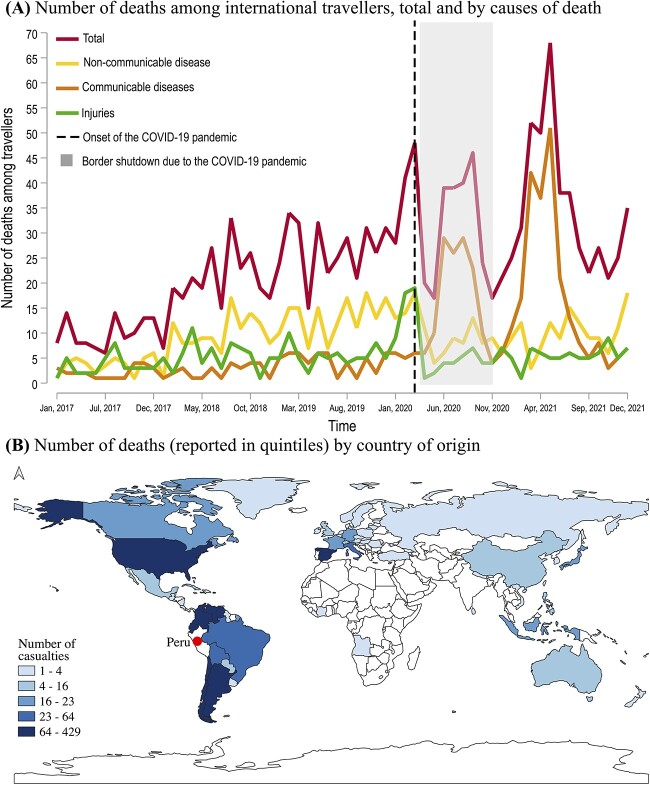
Number of deaths by (A) causes and (B) country of origin of international travellers in Peru from 2017 to 2021. Notes: White areas in (B) stand for countries with no casualties reported among international travellers in Peru. Supplementary Figure A5 shows the exact number of deaths per country of origin. The onset of the COVID-19 pandemic was reported on the 6th of March 2020.

The years with highest incidence of death were 2021 (*n* = 434, 28.7%) and 2020 (*n* = 377, 24.9%). Both years had a large spike in communicable diseases associated deaths caused by COVID-19 (Figure 1A). There were 0.2 deaths per 10 000 travellers in 2017 (Supplementary Figure A2), which gradually increased to 0.5 deaths per 10 000 travellers in 2018, 0.7 deaths per 10 000 travellers in 2019, 4.2 deaths per 10 000 travellers in 2020 and 9.9 deaths per 10 000 travellers in 2021. Compared with 2017, mortality rates were 1.5, 2.5, 20 and 48.5-times greater in 2018, 2019, 2020 and 2021, respectively. Number of deaths among Venezuelan nationals accounted greatly for these differences (i.e. 6, 60, 86, 108 and 169 deaths in this group were reported in 2017, 2018, 2019, 2020 and 2021, respectively). There were monthly differences in adjusted and unadjusted incidence of deaths in Peru (Supplementary Figures A3 and A4). In January 2017, there were 8 deaths (~0.2 deaths per 10 000 travellers), and this number increased to 27 deaths (~1.1 deaths per 10 000 travellers) by January 2020 preceding the COVID-19 pandemic (Supplementary Figures A3 and A4).

Most travellers who died during the study period originated in the Americas (78.9%, *n* = 1196), with a substantial representation from Venezuela (28.3%, *n* = 429), the USA (18.0%, *n* = 273) and Chile (7.0%, *n* = 106) (Figure 1B, and Supplementary Table A4). Europe was the continent of origin for 15.2% (*n* = 230) of the travellers who died, with three quarters of these coming from Spain (5.9%, *n* = 89), Italy (3.8%, *n* = 59) and France (1.4%, *n* = 21). Overall, most travellers came from LMICs (55.9%, *n* = 847) and died in the coastal provinces of Lima (40.9%, *n* = 619) and Callao (6.3%, *n* = 95). Autopsies were conducted on 32% (*n* = 486) of the cases.

### Causes of death among international travellers in Peru

**Figure 2:**
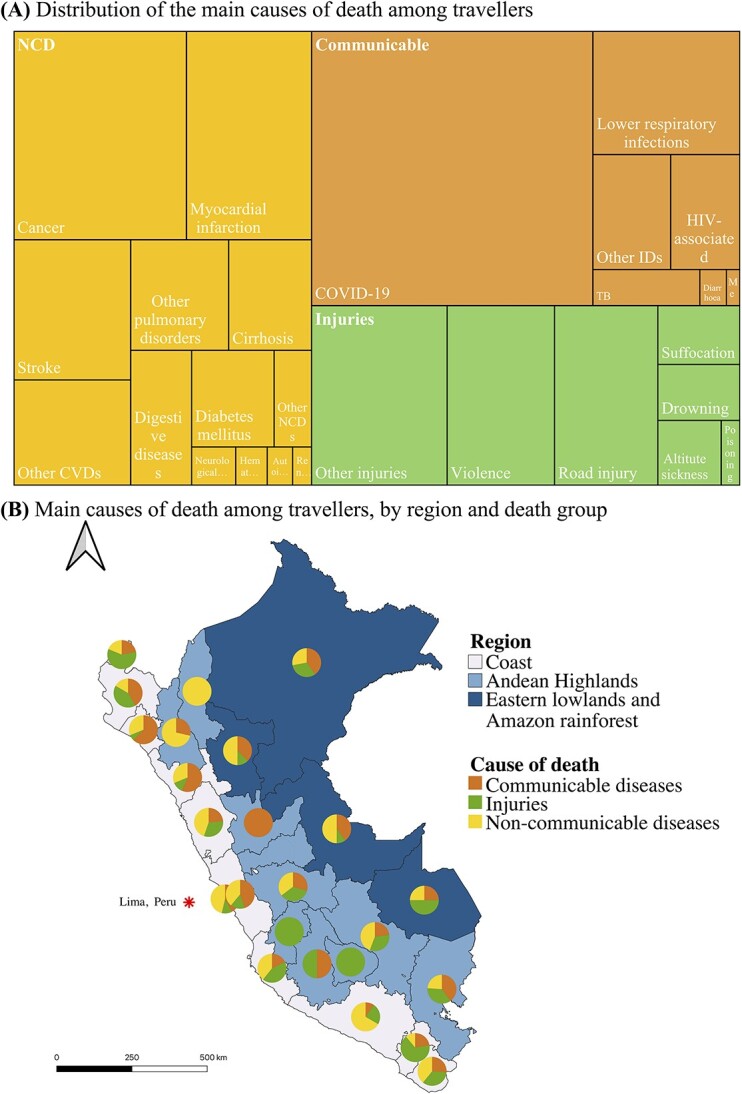
(A) Main causes of death and (B) by geographical distribution among international travellers in Peru from 2017 to 2021, (for 1336 cases with information on causes of death)‡. Notes: Lima, capital city. ‡Deaths classified as ‘unknown’ were excluded from this analysis. In (A), the size of the squares and rectangles reflects the relative proportion of different causes of death. HIV = Human immunodeficiency virus, TB = Tuberculosis, CVD = cardiovascular disease, IDs = Infectious diseases, NCDs = non-communicable diseases. In (B), Geographic Information System Open-Source Geospatial Foundation Project (QGIS) version 2022 was used for map visualization.

The most common causes of death were NCDs (36.9%, *n* = 560), followed by communicable diseases (31.2%, *n* = 487) and injuries (21.1%, *n* = 319) (Table 1 and Supplementary Figure A6). Causes were unknown among 9.8% of deaths. Figure 2A displays disease burden distributions and breakdowns by cause of death. COVID-19 caused 21.1% (*n* = 320) of all the deaths registered since 2020. NCDs, such as cancer (9.8%), myocardial infarction (7.1%) and stroke (4.5%) caused the higher burdens after COVID-19 (Table 1). Non-traffic related trauma (6.3%), road traffic injuries (5.1%) and violence or aggression-associated (5.3%) deaths were the most common among the mortal injuries. If the COVID-19-associated deaths are excluded, the distribution of causes of death was similar to those among Peruvian nationals in 2019, except for a higher prevalence of injury-associated deaths, specifically for violence and other injuries (e.g. traumas) (Supplementary Figures A11 and A12).

**Table 1:** Classification of causes of death among international travellers in Peru from 2017 until 2021 (*N* = 1514)

Cause of death		Number of deaths	Percentage
NCDs	560	36.99	
Cancer	149	9.84	
Myocardial infarction	108	7.13	
Stroke	68	4.49	
Other CVDs	51	3.37	
Other pulmonary disorders	45	2.97	
Asthma	2	0.13	
COPD	3	0.2	
Embolism	13	0.86	
ILD	9	0.59	
Others	18	1.19	
Cirrhosis	38	2.51	
Digestive diseases	34	2.25	
Diabetes mellitus	33	2.18	
Other NCDs	15	0.99	
Neurological diseases	7	0.46	
Haematological diseases	5	0.33	
Autoimmune	4	0.26	
Renal diseases	3	0.2	
Communicable diseases	487	32.16	
COVID-19	320	21.14	
Lower respiratory infections	75	4.95	
Other IDs	37	2.44	
HIV-associated	33	2.18	
Tuberculosis	16	1.06	
Diarrhoea	4	0.26	
Meningitis	2	0.13	
Injuries	319	21.07	
Other injuries	101	6.67	
Electrocution	2	0.13	
Fall	3	0.20	
Other trauma	96	6.34	
Violence	80	5.28	
Gun or knife	51	3.37	
Strangulation or hanging	22	1.45	
Others	7	0.46	
Traffic accidents	77	5.09	
Suffocation	20	1.32	
Drowning	19	1.25	
Altitude sickness	17	1.12	
Poisoning	5	0.33	
Unknown/not determined	148	9.78	

Notes: COPD = chronic obstructive pulmonary disease; ILD = interstitial lung disease; IDs = infectious diseases; HIV = human immunodeficiency virus; CVD = cardiovascular diseases; NCDs = non-communicable diseases.

Monthly mortality stratified by cause of death and adjusted by number of travellers to Peru during the same period increased over time, particularly after the start of the COVID-19 pandemic (Figure 1B and Supplementary Figures A7–A8). Between November 2020 and December 2021(at the peak of the COVID-19 pandemic in Peru), the mortality caused by communicable diseases was 149 times higher than the mortality between January 2017 and April 2020. Similarly, the mortality due to NCD and injuries between November 2020 and December 2021 was 24 and 12 times higher, respectively, compared with the mortality between January 2017 and April 2020 (Supplementary Figures A7–A8). Compared with 2017, the average unadjusted number of deaths increased 380, 240 and 60% between 2018 and 2021 among communicable, NCD and injury-associated deaths, respectively (Supplementary Figures A9–A10).

A high proportion of travellers in the highlands and rainforest died from injuries compared with communicable diseases (*P* = 0.007) and NCDs (*P* = 0.038), respectively (Table 2). While an association between the highland regions and injury-associated mortality was evident, there was no association between the regions were death occurred and mortality associated with communicable diseases or NCD (Figure 2B).

**Table 2:** Characteristics of international travellers in Peru from 2017 to2021, by cause of death (for 1336 with information on causes of death)^a^

Variable	Total	NCD	Communicable	Injuries	χ^2^-test^b^	χ^2^-test^b^	χ^2^-test^b^
	*N* = 1514	*N* = 560	*N* = 487	*N* = 319	C vs I	C vs N	I vs N
	(*n*, %)	(*n*, %)	(*n*, %)	(*n*, %)	*P*-value	*P*-value	*P*-value
Sex at birth							
Male	973 (64.27)	320 (57.1)	300 (61.6)	249 (78.1)	<0.001	0.143	<0.001
Female	541 (35.73)	240 (42.8)	187 (38.4)	70 (21.9)	<0.001	0.143	<0.001
Age group (years)							
<18	39 (2.58)	8 (1.43)	11 (2.26)	14 (4.39)	0.088	0.316	0.007
18–29	203 (13.41)	27 (4.82)	21 (4.31)	130 (40.75)	<0.001	0.695	<0.001
30–39 years	178 (11.76)	33 (5.89)	43 (8.83)	69 (21.63)	<0.001	0.068	<0.001
40–49 years	208 (13.74)	69 (12.32)	65 (13.35)	47 (14.73)	0.578	0.621	0.310
50–59 years	237 (15.65)	100 (17.86)	92 (18.89)	24 (7.52)	<0.001	0.667	<0.001
60–69 years	260 (17.17)	112 (20.00)	110 (22.59)	22 (6.9)	<0.001	0.307	<0.001
70–79 years	202 (13.34)	103 (18.39)	77 (15.81)	9 (2.82)	<0.001	0.269	<0.001
≥80 years	187 (12.35)	108 (19.29)	68 (13.96)	4 (1.25)	<0.001	0.022	<0.001
Marital status							
Divorced or separated	56 (3.74)	24 (4.23)	22 (4.59)	4 (1.27)	0.084	0.221	0.525
Married or in partnership	476 (31.81)	205 (37.00)	159 (33.12)	80 (20.23)	0.413	0.755	0.575
Other not specified	22 (1.47)	50 (9.03)	3 (0.62)	8 (2.52)	0.699	0.595	0.939
Single	728 (48.66)	227 (40.97)	237 (49.38)	189 (59.62)	0.003	0.008	<0.001
Widowed	63 (4.21)	37 (6.68)	19 (3.96)	4 (1.26)	0.027	0.052	<0.001
No information	151 (10.09)	11 (1.98)	40 (8.33)	32 (10.09)	<0.001	0.257	<0.001
Ethnicity							
Afro-American	20 (1.32)	8 (1.43)	0 (0.00)	9 (2.82)	<0.001	0.008	0.149
Asian	52 (3.43)	25 (4.46)	6 (1.23)	10 (3.13)	0.058	0.002	0.333
Mixed	1077 (71.14)	403 (71.96)	429 (88.09)	180 (56.43)	0.174	0.091	0.830
Others^c^	15 (0.99)	8 (1.43)	2 (0.42)	4 (1.25)	<0.001	<0.001	<0.001
No information	350 (3.12)	116 (20.71)	50 (10.27)	116 (36.36)	<0.001	<0.001	<0.001
Year of death							
2017	117 (7.73)	38 (6.79)	22 (4.52)	42 (13.17)	<0.001	0.116	0.002
2018	260 (17.17)	124 (22.14)	30 (6.16)	65 (20.38)	<0.001	<0.001	0.540
2019	326 (21.53)	160 (28.57)	53 (10.88)	70 (21.94)	<0.001	<0.001	0.032
2020	377 (24.9)	115 (20.54)	165 (33.88)	75 (23.51)	0.002	<0.001	0.303
2021	434 (28.67)	123 (21.96)	217 (44.56)	67 (21.00)	<0.001	<0.001	0.739
Season^d^							
Summer (Dec–Mar)	411 (27.13)	140 (25)	138 (28.34)	96 (30.09)	0.592	0.223	0.102
Autumn (Mar–Jun)	400 (26.4)	119 (21.25)	172 (35.32)	66 (20.69)	<0.001	<0.001	0.844
Spring (Sep–Dec)	382 (25.21)	157 (28.04)	109 (22.38)	76 (23.82)	0.634	0.036	0.174
Winter (Jun–Sep)	322 (21.25)	144 (25.71)	68 (13.96)	81 (25.39)	<0.001	<0.001	0.916
Continent of origin							
Africa	3 (0.20)	0 (0.00)	0 (0.00)	2 (0.62)	0.080	-	0.061
America	1196 (78.99)	433 (77.33)	400 (82.13)	265 (83.07)	0.733	0.054	0.043
Asia	75 (5.10)	31 (5.54)	16 (3.29)	12 (3.76)	0.718	0.079	0.242
Europe	230 (15.19)	93 (16.61)	69 (14.17)	37 (11.6)	0.292	0.277	0.044
Oceania	10 (0.66)	3 (0.54)	2 (0.41)	3 (0.94)	0.349	0.770	0.484
Country of origin by income level							
HIC	667 (44.06)	283 (50.4)	223 (45.7)	90 (28.2)	<0.001	0.126	<0.001
LMIC	847 (55.94)	277 (49.5)	264 (54.2)	229 (71.8)	<0.001	0.126	<0.001
Region in Peru^c^							
Highlands	126 (10.24)	41 (9.53)	33 (7.27)	36 (14.88)	<0.001	<0.001	0.072
Coast	1050 (85.31)	371 (86.28)	402 (88.55)	192 (79.34)	0.025	0.732	0.046
Rainforest	55 (4.47)	18 (4.19)	19 (4.19)	14 (5.79)	0.733	0.549	0.372

^a^Deaths classified as ‘Unknown’ were excluded from this analytical sample. vs = versus. I, N and C are for injuries, non-communicable and communicable diseases, respectively.

^b^ *χ* ^2^-tests comparing two categories of causes of deaths (e.g. communicable vs NCDs or communicable vs injuries) at the time across the predictor variables.

^c^Missing data were ~25% of the sample. Other ethnic backgrounds included Amazonian, Aymara and Quechua. HIC = high-income country; LMIC = low- and middle-income country.

^d^Season months are abbreviated in parentheses (e.g. Dec = December, Jun = June, Mar = March, Sep = September).

**Figure 3:**
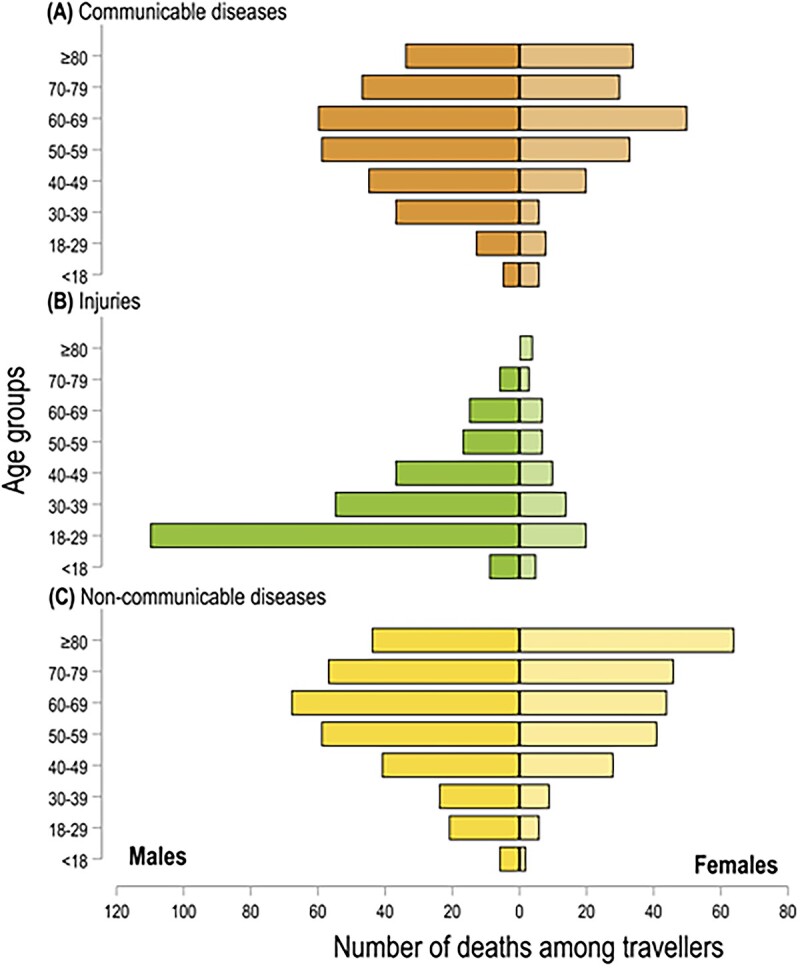
Distribution of deaths by age-group and sex for (A) communicable diseases, (B) injuries and (C) NCDs among international travellers in Peru from 2017 to 2021(for 1336 cases with information on causes of death)‡. Notes: ‡Deaths classified as ‘Others’ were excluded from this analysis. The vertical black line on the *x*-axis separates the sample by sex (i.e. males and females).

Among injury-associated deaths, most travellers were males (78.1%). The proportion of males among injury-associated deaths was greater compared with other death groups (57.1 and 61.6% among NCDs and communicable diseases, respectively, *P* < 0.001) (Table 2). Young adults aged 18–29 years had a significantly higher chance of dying from injuries (40.8%, compared with 4.8 and 4.3% for NCDs and communicable diseases, respectively, within the same age group). Deaths in the 60–69 years age group were predominantly associated with communicable diseases (22.6%) or NCDs (20.0%) compared with injuries (6.9%, *P*-value<0.001) (Figure 3). Travellers who died from injuries were predominantly single (59.6%), a significantly higher proportion (χ^2^ -test *P*-value < 0.001), relative to those associated with communicable diseases (49.4%) and NCDs (40.9%). Marital status groups displayed no significant disparity between deaths associated with communicable diseases and those due to NCDs (χ^2^ -test *P*-value > 0.05). Among communicable diseases, there were considerable differences between year of death and season, mostly due to the COVID-19 pandemic. Country of origin was not associated with cause of death, although injury-associated casualties were seen in a greater proportion of travellers from LMICs (71.8%, compared with 54.2 and 49.5% among communicable and NCDs, respectively (χ^2^ -test *P*-value < 0.001).

### Deaths where risks could potentially have been prevented or reduced by pre-travel advice

Finally, for 873 (57.7%) of deaths, the risk could potentially have been attenuated thorough comprehensive pre-travel advice. These fatalities were predominantly attributable to communicable diseases and injuries. In contrast, using OECD standards, 76.5% could have been avoided through effective public policy intervention at the country of origin (Supplementary Figures A13–A15).

## Discussion

This study provides a comprehensive examination of the number and causes of death amongst international travellers to Peru. We demonstrated an upward trend in the adjusted death incidence between 2017 and 2021 with NCDs identified as the predominant cause of mortality. Communicable diseases were the second most common cause of death and a significant proportion of these were attributed to COVID-19. Substantial heterogeneity in the distribution of causes of death was evident across sociodemographic and geographic domains. Similar patterns were observed among causes of death in Peruvian nationals, except for injury-associated mortality that was more prevalent in international travellers.^29^

The epidemiology of deaths among international travellers to Peru was similar to that reported in studies evaluating deaths of international travellers to and from HIC such as Australia, Canada, Scotland and the USA.^30–34^ Males were more likely to die, consistent with two studies estimating 66 and 65% of total traveller deaths in Australia and the USA were among males, respectively.^30^^,^^31^ CVDs (e.g. ischaemic heart diseases) and cancers were the major causes of death found in our study. One study evaluating travellers to Australia found that 73% of deaths in tourists were associated with natural causes such as ischaemic coronary events and cancer.^31^ In contrast with our study that included a large number of young and Latin American travellers, the majority of the tourists in that study was older and came from HICs such as New Zealand and the UK. In our study, travellers from the USA died most often from NCDs (48%, *n* = 133) at an average age of 70 years and travellers from Venezuela died most often from communicable diseases (36.4%, *n* = 156) at an average age of 44 years. Travellers aged 60–69 had the highest risk of dying, consistent with previous studies.^31^^,^^34–38^ We found that 41.6% of cancer deaths occurred in traveller’s aged <60 years, which was likely to be related to be premature mortality. Difficulties associated with distance or access to treatment, travel stress and lack of knowledge about the health care system might hamper adequate care of travellers diagnosed with cancer.^39^^,^^40^

Communicable diseases were the second most common cause of death in our study. A study of international travellers to the USA before the COVID-19 pandemic showed that 12% of deaths in this group were attributable to IDs.^30^ Our study would have shown similar results if COVID-19-associated mortality was not included in the analysis (167/1046, 15.9%). Forty one percent of COVID-19-associated deaths occurred in 2020 when no medications or vaccines were available and the Peruvian health system collapsed. Most of these deaths were likely to have occurred among expatriates, migrants and refugees. The first COVID-19 vaccines were available in December 2020 but access for travellers, particularly among those from LMIC, and rollout in the Peruvian population occurred much later during the pandemic.^41^ The second cause of communicable diseases mortality was lower respiratory infections, predominantly pneumonia, which aligns with the epidemiological trends observed among Peru’s local population.^42^ Many lower respiratory infections are vaccine-preventable (pneumococcal, respiratory syncytial virus and influenza vaccines), and mortality can be reduced with early appropriate treatment. These findings highlight the importance of ensuring that travellers are up to date with their age-appropriate vaccines for respiratory infections.

Our results showed that injuries accounted for 20% of deaths among travellers to Peru. Similar rates have been documented for travellers from Scotland (20%) and individuals visiting Australia (23%).^31^^,^^32^ Traffic accidents were a common cause of death. In Bermuda, the incidence rate of traffic accidents among international travellers was 94.1 per 1000 person-years compared with the rate of 16.6 among local residents.^43^ Similarly, in Greece, international travellers accounted for 33% of all road traffic accident fatalities.^44^ Different driving regulations and customs may play a role in traffic accident among travellers. Driving on the left side of the road in Australia has been indicated as a risk factor for traffic accidents from countries that drive on the right side.^45^ However, we did not find information that allowed us to infer that travellers were driving when traffic accidents occurred. A global study suggested that travellers aged 20–29 years are at highest risks of dying in accidents accounting for up to 23% of total fatalities in tourists.^38^ Almost half of the deaths reported in that study occurred in Asian countries, where traffic accidents and drowning fatalities are common. In our study, trauma, traffic accidents and violent deaths were common but drowning was not. Young travellers may be victims of crime more often, particularly in larger cities such as Lima, Cusco and Arequipa where tourists may be targeted.^46^ Preventing violent deaths due to crime among travellers should be considered in pre-travel advice and follow international safety guidelines.^47^ In one study in Australia, the highest proportion of injury-associated deaths (51%) was described in international travellers between 15 and 35 years, compared with other age groups.^31^ It is possible that younger travellers engage in riskier activities or exhibit risk taking behaviour (e.g. not wearing seat belts, abusing alcohol) that result in higher mortality associated with injuries compared with older groups. Other factors such as marital status, education and international health insurance coverage could be contributing factors to risk taking behaviour.^48^^,^^49^ It is crucial to develop prevention strategies targeting young travellers to specific regions likely to engage in activities linked to elevated physical hazards.^9^ This should not only include road accidents, but also environmental hazards including drowning, suffocation and altitude related illnesses present in popular travel destinations in Peru such as Lima and Cuzco.^27^ It is imperative to enhance the pre-travel counselling of young people to increase awareness of safety and violence associated injuries.

The results of this study must be interpreted with caution considering the source of the data and epidemiologic situations during the study period. Our study relied on causes of death documentation performed by physicians and other health personnel from different parts of Peru which in some cases could have been incomplete or inaccurate. Our dataset included all non-residents who died in Peru, which would include different types of foreigners. The proportion of tourists, travellers visiting friends and relatives, expatriates, refugees and migrants in our study population was unknown. According to the Peruvian government statistics, Peru hosts the second largest population of Venezuelan migrants and refugees in Latin America with 1.5 million as of August 2023.^50^^,^^51^ However, it is estimated that those figures only account for 70% of the Venezuelans living and working in Peru. Travellers from Venezuela are overrepresented in our sample and are likely responsible for some of the associations described. The economic and migratory status of travellers might also impact on mortality posing significant challenges with social and economic implications for the health system.^52^ Additionally, the influx of migrants and refugees through illegal border crossings may decrease the accuracy of the cumulative death incidence calculations.^53^ The onset of the COVID-19 pandemic influenced the epidemiology of deaths in Peru, increasing numbers attributable to IDs. Seriously ill travellers might have been evacuated to the capital or other metropolitan areas for more complex medical care. This could have resulted in underestimation of the actual death burden in certain regions of Peru. Finally, causes of death were missing for ~10% of cases. Unregistered deaths could be a problem specially among refugee and marginalized populations. Finally, in Peru, reports of missing travellers, which are often presumed death, are not officially included in the statistics. Our study represents a first look at the mortality of international travellers in Peru and further studies addressing specific populations will be needed to inform prevention strategies, and considering longer (>5 years) observation periods will be needed to inform prevention strategies.

## Conclusions

Causes of death among international travellers to Peru mirror the causes of death among travellers to other destinations with some exceptions reflecting the characteristics of our study population. Mortality increased during the study period, even before the COVID-19 pandemic started, which could be related to changing demographics of international arrivals to Peru. The mortality due to COVID-19, especially during border closures, was likely driven by expatriates, migrants and refugees. The risk for most causes of death among travellers to Peru could be decreased by optimizing medical management of chronic diseases and effective pre-travel and preventive advice and interventions for some communicable diseases and injuries. As the travel industry recovers in Peru, the absolute numbers of deaths among travellers will likely increase. Increasing travel medicine practitioners’ awareness of the problem and enhancing emergency and other medical services outside Lima may prevent this trend and improve outcomes. Additionally, it is crucial to consider and address specific risks associated with geographic destinations, such as highlands, rainforest and coastal areas. Future studies should address the root causes leading to death and the risks among different traveller groups in Peru to inform pre-travel preparations.

## Supplementary Material

BeyondtheJourneyPeru_SM_taad163

## Data Availability

Data were retrieved from the Peruvian government records through free access to public information law. These data are freely available upon request at https://www.minsa.gob.pe/defunciones/. Geographic information system (GIS) coordinates and shapefiles are freely available at https://www.geogpsperu.com/2019/08/limite-departamental-politico-shapefile.html.

## CRediT author Statement

Kasim Allel (Conceptualization [supporting], Data curation [equal], Formal analysis [lead], Investigation [equal], Methodology [lead], Project administration [lead], Resources [lead], Software [lead], Validation [equal], Visualization [lead], Writing—original draft [lead], Writing—review & editing [lead]), Miguel Cabada (Conceptualization [equal], Data curation [equal], Formal analysis [equal], Investigation [equal], Methodology [equal], Project administration [equal], Supervision [equal], Validation [equal], Writing—review & editing [equal]), Colleen Lau (Conceptualization [supporting], Supervision [supporting], Writing—review & editing [supporting]), Deborah Mills (Writing—review & editing [equal]), Richard Franklin (Supervision [equal], Visualization [equal], Writing—review & editing [equal]), Yan Zhu (CRediT contribution not specified), and Luis Furuya-Kanamori (Conceptualization [lead], Data curation [equal], Formal analysis [equal], Funding acquisition [equal], Investigation [equal], Methodology [equal], Project administration [equal], Resources [lead], Supervision [equal], Validation [equal], Writing—review & editing [supporting]).
