# Supplementary material for: Causes of death among international travellers in Peru, 2017 to 2021
Source: J Travel Med. 2023 Dec 21;31(1):taad163. doi: 10.1093/jtm/taad163 (PMC10823482; doi:10.1093/jtm/taad163)
Supplement: BeyondtheJourneyPeru_SM_taad163 [file beyondthejourneyperu_sm_taad163.pdf]

Supplementary material

Causes of Death among International Travellers in Peru, 2017 to 2021

Table of Contents

Figure A1. Death certificate [in Spanish] 2

Table A1. Joint OECD/Eurostat lists of preventable and treatable causes of mortality 3

Table A2. Summary table to homologate avoidable and unavoidable deaths using OECD standards and application to travel medicine. 11

Table A3. Demographics and socioeconomic characteristics among dead travellers in Peru (N=1,514) between 2017 and 2021 13

Table A4. Country of origin and location of fatalities among dead travellers in Peru (N=1,514) between 2017 and 2021 15

Figure A2. Total number of deaths over time adjusted to national incidence of travellers in Peru, by year 17

Figure A3. Number of deaths among travellers adjusted to total number of travellers in Peru between 2017 and 2021, by month. 18

Figure A4. Monthly and annual percentage variation in the number of total deaths, respect to the month or year from the reference year 2017. 19

Figure A5. Number of deaths among travellers in Peru, by country origin 20

Figure A7. Number of injuries-associated deaths among travellers adjusted to total number of travellers in Peru between 2017 and 2021, by month 21

Figure A8. Number of non-communicable diseases-associated deaths among travellers adjusted to total number of travellers in Peru between 2017 and 2021, by month 22

Figure A9. Monthly percentage variation in the number of total deaths, respect to the month from the reference year 2017, by death group 23

Figure A10. Annual percentage variation in the number of total deaths, respect to the reference year 2017, by death group 24

Figure A11. Causes of death among travellers accumulated overtime (2017-2021) excluding COVID-19-associated deaths in Peru, by death classification (N= 1, 366)† 25

Figure A12. Distribution of the causes of death in Peru in both sexes and across all ages in 2019 according to the estimates from the Global Burden of Disease 26

Figure A13. Main causes of death among travellers in Peru, by region and death classification using avoidable and non-avoidable mortality 27

Figure A14. Main causes of death among travellers in Peru over time, by death classification 28

Figure A15. Main causes of death among travellers in Peru over time, by death classification OECD!!!!29

Table A5. STROBE guidelines for observational studies 30

References 34

Last update: 4th of October 2023.

Figure A1. Death certificate [in Spanish]

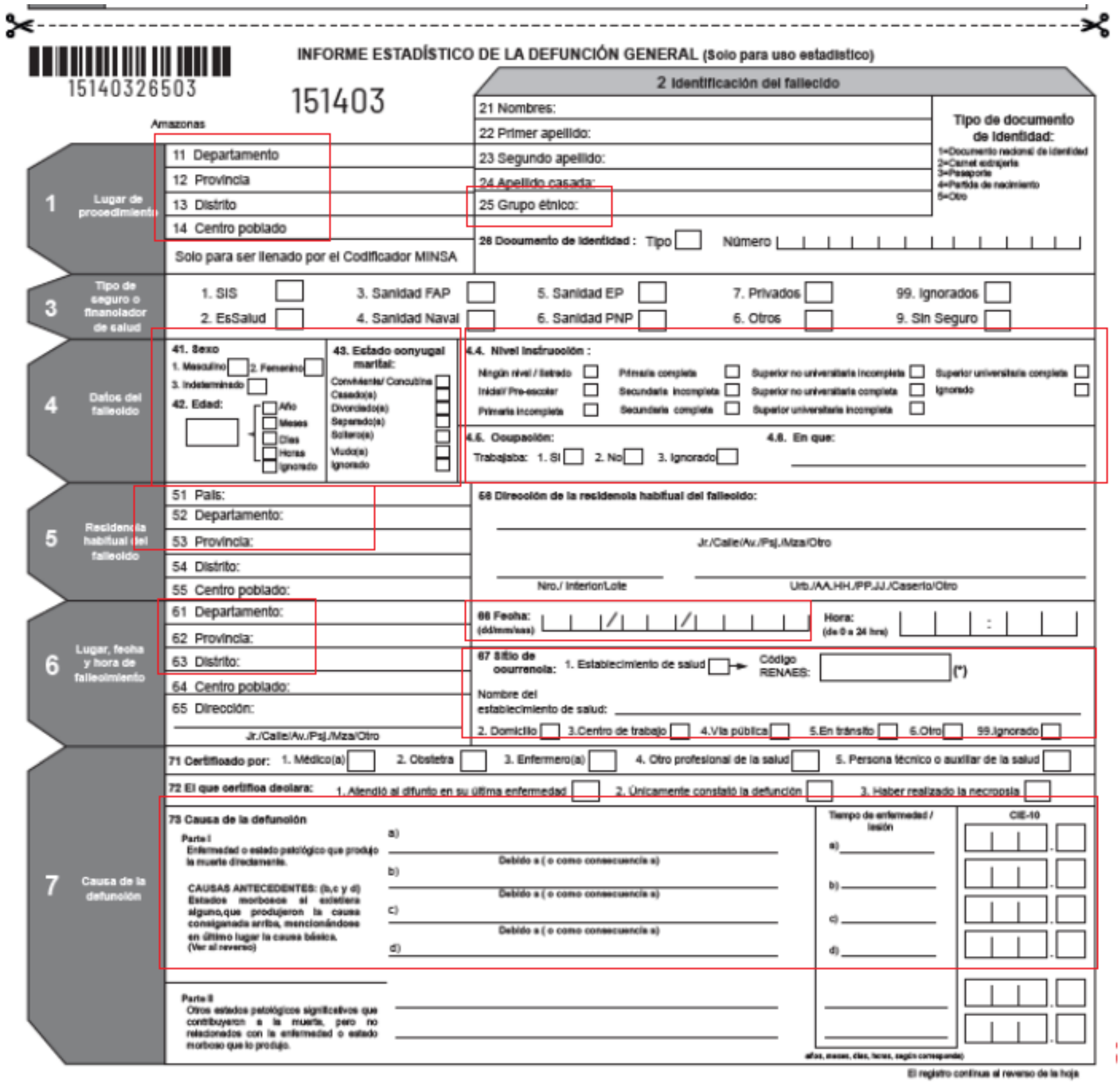

INFORME ESTADÍSTICO DE LA DEFUNCIÓN GENERAL (Solo para uso estadístico)

15140326503

151403

Amazonas

1 Lugar de procedimiento
11 Departamento
12 Provincia
13 Distrito
14 Centro poblado
Solo para ser llenado por el Codificador MINSA

2 Identificación del fallecido
21 Nombres:
22 Primer apellido:
23 Segundo apellido:
24 Apellido casada:
25 Grupo étnico:
Tipo de documento de identidad:
1=Documento nacional de identidad
2=Carnet extranjería
3=Pasaporte
4=Partida de nacimiento
5=Otro
26 Documento de identidad : Tipo Número

3 Tipo de seguro o financiador de salud
1. SIS
2. EsSalud
3. Sanidad FAP
4. Sanidad Naval
5. Sanidad EP
6. Sanidad PNP
7. Privados
8. Otros
99. Ignorados
9. Sin Seguro

4 Datos del fallecido
41. Sexo
1. Masculino 2. Femenino
3. Indeterminado
42. Edad: Año Meses Días Horas Ignorado
43. Estado conyugal marital:
Conviviente/ Concubina
Casado(a)
Divorciado(a)
Separado(a)
Soltero(a)
Viudo(a)
Ignorado
44. Nivel instrucción :
Ningún nivel / Iletrado
Inicial/ Pre-escolar
Primaria incompleta
Primaria completa
Secundaria incompleta
Secundaria completa
Superior no universitaria incompleta
Superior no universitaria completa
Superior universitaria incompleta
Superior universitaria completa
Ignorado
45. Ocupación:
Trabajaba: 1. Si 2. No 3. Ignorado
46. En que:

5 Residencia habitual del fallecido
51 País:
52 Departamento:
53 Provincia:
54 Distrito:
55 Centro poblado:
56 Dirección de la residencia habitual del fallecido:
Jr./Calle/Av./Psj./Mza/Otro
Nro./ Interior/Lote
Urb./AA.HH./PP.JJ./Caserío/Otro

6 Lugar, fecha y hora de fallecimiento
61 Departamento:
62 Provincia:
63 Distrito:
64 Centro poblado:
65 Dirección:
Jr./Calle/Av./Psj./Mza/Otro
66 Fecha: (dd/mm/aaa)
Hora: (de 0 a 24 hrs)
67 Sitio de ocurrencia: 1. Establecimiento de salud
Código RENAES: (*)
Nombre del establecimiento de salud:
2. Domicilio 3.Centro de trabajo 4.Vía pública 5.En tránsito 6.Otro 99.Ignorado

7 Causa de la defunción
71 Certificado por: 1. Médico(a) 2. Obstetra 3. Enfermero(a) 4. Otro profesional de la salud 5. Persona técnico o auxiliar de la salud
72 El que certifica declara: 1. Atendió al difunto en su última enfermedad 2. Únicamente constató la defunción 3. Haber realizado la necropsia
73 Causa de la defunción
Parte I
Enfermedad o estado patológico que produjo la muerte directamente.
CAUSAS ANTECEDENTES: (b,c y d) Estados morbosos si existiera alguno,que produjeron la causa consignada arriba, mencionándose en último lugar la causa básica. (Ver al reverso)
a)
Debido a (o como consecuencia a)
b)
Debido a (o como consecuencia a)
c)
Debido a (o como consecuencia a)
d)
Parte II
Otros estados patológicos significativos que contribuyeron a la muerte, pero no relacionados con la enfermedad o estado morboso que lo produjo.
Tiempo de enfermedad / lesión
a)
b)
c)
d)
años, meses, días, horas, según corresponda)
CIE-10
El registro continua al reverso de la hoja

Table A1. Joint OECD/Eurostat lists of preventable and treatable causes of mortality

Group	Causes of deaths	Preventable mortality	Treatable mortality	ICD-10 Code	Age threshold	Rationale for inclusion
Infectious diseases	Intestinal diseases	x		A00-A09	0-74	Most of these infections can be prevented through prevention measures (e.g. improve water and food safety)
	Diphtheria, Tetanus, Poliomyelitis	x		A35, A36, A80	0-74	Most of these infections can be prevented through vaccination.
	Whooping cough	x		A37	0-74	Most of these infections can be prevented through vaccination.
	Meningococcal infection	x		A39	0-74	Most of these infections can be prevented through vaccination.
	Sepsis due to streptococcus pneumonia and depsis due to hemophilus influenzae	x		A40.3, , A41.3	0-74	Most of these infections can be prevented through vaccination.
	Haemophilus inflenza infections	x		A49.2	0-74	Most of these infections can be prevented through vaccination.
	Sexually transmitted infections (except HIV/AIDS)	x		A50-A60, A63, A64	0-74	These infections can be prevented through prevention measures.
	Varicella	x		B01	0-74	Most of these infections can be prevented through vaccination.
	Measles	x		B05	0-74	Most of these infections can be prevented through vaccination.
	Rubella	x		B06	0-74	Most of these infections can be prevented through vaccination.
	Viral Hepatitis	x		B15-B19	0-74	This condition is preventable and will not require treatment if prevented.
	HIV/AIDS	x		B20-B24	0-74	This condition is preventable and will not require treatment if prevented.
	Malaria	x		B50-B54	0-74	This condition is preventable and will not require treatment if prevented.
	Haemophiles and pneumococcal meningitis	x		G00.0, G00.1	0-74	Most of these infections can be prevented through vaccination.
	Tuberculosis	x (50%)	x (50%)	A15-A19, B90, J65	0-74	Reduction in deaths from tuberculosis in several countries has been about evenly achieved through greater prevention (reduction in incidence) and earlier detection and more effective treatment (higher survival rates).

	Scarlet fever		x	A38	0-74	Case-fatality rates can be reduced through early detection and appropriate antibiotic treatment.
	Sepsis		x	A40 (excl. A40.3),A41 (excl. A41.3)	0-74	Case-fatality rates can be reduced through greater quality of care and reduced patient adverse events, and early detection and appropriate antibiotic treatment.
	Cellulitis		x	A46, L03	0-74	Case-fatality rates can be reduced through early detection and appropriate antibiotic treatment.
	Legionnaires disease		x	A48.1	0-74	Case-fatality rates can be reduced through early detection and appropriate antibiotic treatment.
	Streptococcal and enterococci infection		x	A49.1	0-74	Case-fatality rates can be reduced through early detection and appropriate antibiotic treatment.
	Other meningitis		x	G00.2, G00.3, G00.8, G00.9	0-74	Case-fatality rates can be reduced through early detection and appropriate antibiotic treatment.
	Meningitis due to other and unspecified causes		x	G03	0-74	Case-fatality rates can be reduced through early detection and appropriate antibiotic treatment.
Cancer	Lip, oral cavity and pharynx cancer	x		C00-C14	0-74	This condition can be largely prevented through prevention measures (e.g. reduce smoking).
	Oesophageal cancer	x		C15	0-74	This condition can be largely prevented through prevention measures (e.g. reduce smoking).
	Stomach cancer	x		C16	0-74	This condition can be largely prevented through prevention measures (e.g. reduce smoking and alcohol consumption, and improve nutrition).
	Liver cancer	x		C22	0-74	This condition can be largely prevented through prevention measures (e.g. reduce smoking and alcohol consumption).
	Lung cancer	x		C33-C34	0-74	This condition can be largely prevented through prevention measures (e.g. reduce smoking).
	Mesothelioma	x		C45	0-74	This condition can be largely prevented through prevention measures (e.g. reduce asbestos exposure).
	Skin (melanoma) cancer	x		C43	0-74	This condition can be largely prevented through prevention measures (e.g. reduce sun exposure).

	Bladder cancer	x		C67	0-74	This condition can be largely prevented through prevention measures (e.g. reduce smoking).
	Cervical cancer	x (50%)	x (50%)	C53	0-74	Cervical cancer can be prevented through vaccination and screening can also find pre-cancerous abnormalities that can be treated to prevent cancer, but five-year survival after cancer detection is also relatively high and rising.
	Colorectal cancer		x	C18-C21	0-74	Case-fatality rates have been reduced through earlier detection and treatment. Five-year survival after detection is relatively high and rising.
	Breast cancer (female only)		x	C50	0-74	Case-fatality rates have been reduced through earlier detection and treatment. Five-year survival after detection is relatively high and rising.
	Uterus cancer		x	C54,C55	0-74	Case-fatality rates have been reduced through earlier detection and treatment. Five-year survival after detection is relatively high and rising.
	Testicular cancer		x	C62	0-74	Case-fatality rates have been reduced through earlier detection and treatment. Five-year survival after detection is relatively high and rising.
	Thyroid cancer		x	C73	0-74	Case-fatality rates have been reduced through early detection and appropriate treatment.
	Hodgkin's disease		x	C81	0-74	Case-fatality rates have been reduced through early detection and appropriate treatment.
	Lymphoid leukaemia		x	C91.0, C91.1	0-74	Case-fatality rates have been reduced through early detection and appropriate treatment.
	Benign neoplasm		x	D10-D36	0-74	Case-fatality rates have been reduced through early detection and appropriate treatment.

Endocrine and metabolic diseases	Nutritional deficiency anaemia	x		D50-D53	0-74	This condition can be largely prevented through prevention measures (e.g. improve nutrition).
	Diabetes mellitus	x (50%)	x (50%)	E10-E14	0-74	Type 1 diabetes is not preventable, but appropriate treatments can reduce mortality. Type 2 diabetes is largely preventable (e.g. improve nutrition), but appropriate treatments can also reduce mortality.
	Thyroid disorders		x	E00-E07	0-74	Case-fatality rates can be reduced through early detection and appropriate treatment.
	Adrenal disorders		x	E24-E25 (except E24.4), E27	0-74	Case-fatality rates can be reduced through early detection and appropriate treatment.
Diseases of the nervous system	Epilepsy		x	G40,G41	0-74	Case-fatality rates can be reduced through early detection and appropriate treatment.
Diseases of the circulatory system	Aortic aneurysm	x (50%)	x (50%)	I71	0-74	This condition is both preventable through prevention measures (similar risk factors as for ischaemic heart diseases) and treatable.
	Hypertensive diseases	x (50%)	x (50%)	I10-I13, I15	0-74	This condition is both preventable through prevention measures (e.g. reduce smoking, improve nutrition and physical activity) and treatable.
	Ischaemic heart diseases	x (50%)	x (50%)	I20-I25	0-74	Reduction in deaths from IHD over the past decades in several countries has been about evenly achieved through greater prevention (reduction in incidence) and earlier detection and more effective treatment (higher survival rates).
	Cerebrovascular diseases	x (50%)	x (50%)	I60-I69	0-74	Reduction in deaths from CVD over the past decades in several countries has been about evenly achieved through greater prevention (reduction in incidence) and earlier detection and more effective treatment (higher survival rates).
	Other atherosclerosis	x (50%)	x (50%)	I70, I73.9	0-74	This condition is both preventable through prevention measures (e.g. improve nutrition) and treatable.
	Rheumatic and other heart disease		x	I00-I09	0-74	Case-fatality rates can be reduced through appropriate treatment.

	Venous thromboembolism		x *	I26, I80, I82.9	0-74	The majority of venous thrombosis events result from hospitalisations. These cases are treatable to the extent that they are linked to the quality of care that people receive.
Diseases of the respiratory system	Influenza	x		J09-J11	0-74	Most of the deaths can be prevented through prevention measures (e.g. vaccination).
	Pneumonia due to Streptococcus pneumonia or Haemophilus influenza	x		J13-J14	0-74	Most of these infections can be prevented through vaccination.
	Chronic lower respiratory diseases	x		J40-J44	0-74	This condition can be largely prevented through prevention measures (e.g. reduce smoking).
	Lung diseases due to external agents	x		J60-J64, J66-J70, J82, J92	0-74	This condition can be largely prevented through prevention measures (e.g. reduce exposure to chemical, gases and other agents).
	Upper respiratory infections		x	J00-J06, J30-J39	0-74	Case-fatality rates can be reduced through appropriate treatment.
	Pneumonia, not elsewhere classified or organism unspecified		x	J12, J15, J16- J18	0-74	Case-fatality rates can be reduced through early detection and appropriate antibiotic treatment.
	Acute lower respiratory infections		x	J20-J22	0-74	Case-fatality rates can be reduced through appropriate treatment.
	Asthma and bronchiectasis		x	J45-J47	0-74	Case-fatality rates can be reduced through appropriate treatment (e.g. medication).
	Adult respiratory distress syndrome		x	J80	0-74	Case-fatality rates can be reduced through appropriate treatment.
	Pulmonary oedema		x	J81	0-74	Case-fatality rates can be reduced through appropriate treatment.
	Abscess of lung and mediastinum pyothorax		x	J85, J86	0-74	Case-fatality rates can be reduced through appropriate treatment.
	Other pleural disorders		x	J90, J93, J94	0-74	Case-fatality rates can be reduced through appropriate treatment.
Diseases of the digestive system	Gastric and duodenal ulcer		x	K25-K28	0-74	Case-fatality rates can be reduced through early detection and appropriate treatment..
	Appendicitis		x	K35-K38	0-74	Case-fatality rates can be reduced through early detection and appropriate treatment.
	Abdominal hernia		x	K40-K46	0-74	Case-fatality rates can be reduced through early detection and appropriate treatment.

	Cholelithiasis and cholecystitis		x	K80-K81	0-74	Case-fatality rates can be reduced through early detection and appropriate treatment.
	Other diseases of gallbladder or biliary tract		x	K82-K83	0-74	Case-fatality rates can be reduced through early detection and appropriate treatment.
	Acute pancreatitis		x	K85.0,1,3,8,9	0-74	Case-fatality rates can be reduced through early detection and appropriate treatment.
	Other diseases of pancreas		x	K86.1,2,3,8,9	0-74	Case-fatality rates can be reduced through early detection and appropriate treatment.
Diseases of the genitourinary system	Nephritis and nephrosis		x	N00-N07	0-74	Case-fatality rates can be reduced through early detection and appropriate treatment.
	Obstructive uropathy		x	N13,N20-N21, N35	0-74	Case-fatality rates can be reduced through early detection and appropriate treatment.
	Renal failure		x	N17-N19	0-74	Case-fatality rates can be reduced through early detection and appropriate treatment.
	Renal colic		x	N23	0-74	Case-fatality rates can be reduced through early detection and appropriate treatment.
	Disorders resulting from renal tubular dysfunction		x	N25	0-74	Case-fatality rates can be reduced through early detection and appropriate treatment.
	Unspecified contracted kidney, small kidney of unknown cause		x	N26-N27	0-74	Case-fatality rates can be reduced through early detection and appropriate treatment.
	Inflammatory diseases of genitourinary system		x	N34.1,N70-N73,N75.0,N75.1,N76.4,6	0-74	Case-fatality rates can be reduced through early detection and appropriate treatment.
	Prostatic hyperplasia		x	N40	0-74	Case-fatality rates can be reduced through early detection and appropriate treatment.
Pregnancy, childbirth and perinatal period	Tetanus neonatorum	x		A33	0-74	Most of these infections can be prevented through vaccination.
	Obstetrical tetanus	x		A34	0-74	Most of these infections can be prevented through vaccination.
	Pregnancy, childbirth and the puerperium		x	O00-O99	0-74	Effective treatment is available in most cases to avoid maternal mortality.
	Certain conditions originating in the perinatal period		x	P00-P96	0-74	Case-fatality rates can be reduced through early detection and appropriate treatment.

Congenital malformations	Certain congenital malformations (neural tube defects)		x		Q00, Q01, Q05	0-74	These conditions can be prevented through prevention measures (improve maternal nutrition, e.g. folic acid consumption).
	Congenital malformations of the circulatory system (heart defects)			x	Q20-Q28	0-74	These conditions can be treated through surgical operations
Adverse effects of medical and surgical care	Drugs, medicaments andbiological substances causing adverse effects in therapeutic use			x *	Y40-Y59	0-74	These conditions are treatable through better drug prescription and adherence.
	Misadventures to patients during surgical and medical care			x *	Y60-Y69,Y83-Y84	0-74	These conditions are treatable through better quality of care that patients receive.
	Medical devices associated with adverse incidents in diagnostic and therapeutic use			x *	Y70–Y82	0-74	These conditions are treatable through better quality of care that patients receive.
Injuries	Transport Accidents		x		V01-V99	0-74	Deaths can be prevented through public health interventions (e.g. road safety measures).
	Accidental Injuries		x		W00-X39, X46-X59	0-74	Deaths can be prevented through public health interventions (e.g. injury prevention campaigns).
	Intentional self-harm		x		X66-X84	0-74	Deaths can be prevented through public health interventions (e.g. suicide prevention campaigns).
	Event of undetermined intent		x		Y16-Y34	0-74	Deaths can be prevented through public health interventions (e.g. harm prevention campaigns).
	Assault		x		X86-Y09	0-74	Deaths can be prevented through public health interventions.
Alcohol-related and drug-related deaths	Alcohol-related deaths	Alcohol-specific disorders and poisonings	x		E24.4, F10, G31.2, G62.1, G72.1, I42.6, K29.2, K70, K85.2, K86.0, Q86.0, R78.0, X45, X65, Y15	0-74	Deaths can be largely prevented through public health interventions (e.g. alcohol control policies).
		Other alcohol-related disorders	x		K73, K74.0-K74.2, K74.6	0-74	Deaths can be largely prevented through public health interventions (e.g. alcohol control policies).
	Drug-related deaths**	Drug disorders and poisonings	x		F11-F16, F18-F19, X40-X44, X85, Y10-Y14	0-74	Deaths can be largely prevented through public health interventions (e.g. drug control policies).
		Intentional self-poisoning by drugs	x		X60-X64	0-74	Deaths can be largely prevented through public health interventions (e.g. drug control policies).
Provisional assignment of new diseases	COVID-19		x		U07.1- U07.2	0-74	Most of these infections and deaths can be prevented through prevention measures (including vaccination).

Notes: Table extracted from OECD/Eurostat report (1). Avoidable mortality considered those deaths that could be prevented, treated or both. Preventable deaths are those avoided trough effective public health or interventions, whereby treatable can be avoided through treatment.

Table A2. Summary table to homologate avoidable and unavoidable deaths using OECD standards and application to travel medicine.

	**Standard definition off avoidable and non-avoidable deaths (OECD)**				**Risks that could be reduced through pre-travel advice**	
Cause of death	Preventable	Treatable	Preventable & treatable	Non-avoidable	Preventable OR modifiable	Non-preventable OR modifiable
**Communicable**						
COVID-19	X				X	
Diarrhoea	X				X	
HIV-associated	X				X	
Lower respiratory infections	X				X	
Meningitis		X			X	
Other IDs	X				X	
Tuberculosis			X		X	
**Injuries**						
Altitude sickness	X				X	
Drowning	X				X	
Other injuries	X				X	
Poisoning	X				X	
Road injury	X				X	
Suffocation	X				X	
Violence	X				X	
**NCD**						
Autoimmune				X		X

Cancer ✶	X	X	X			X
Cirrhosis	X					X
Diabetes mellitus			X		X	
Digestive diseases		X			X	
Haematological diseases			X			X
Myocardial infarction			X			X
Neurological diseases			X			X
Other CVDs			X			X
Other NCDs ✶	X	X	X			X
Other pulmonary disorders ✶	X	X				X
Renal diseases		X				X
Stroke			X			X

Notes: OECD= Organisation for Economic Cooperation and Development. NCD= non-communicable diseases. CVD= cardiovascular diseases. IDs= Infectious diseases. HIV= human immunodeficiency virus. ✢ Depending upon each specific health condition, it could be classified in different ways.

Table A3. Demographics and socioeconomic characteristics among dead travellers in Peru (N=1,514) between 2017 and 2021

Variable name	Percentage	Number of observations
Sex at birth (%, n)		
Male	64.27	973
Female	35.73	541
Age group (%, n)		
<18 years	2.58	39
18-29 years	13.41	203
30-39 years	11.76	178
40-49 years	13.74	208
50-59 years	15.65	237
60-69 years	17.17	260
70-79 years	13.34	202
≥80 years	12.35	187
Marital status (%, n)		
Divorced	3.54	53
In partnership	2.01	30
Married	29.81	446
Other	1.47	22
Separated	0.2	3
Single	48.66	728
Widowed	4.21	63
Not answered	10.09	151
Ethnicity (%, n)		
Afro-American	1.32	20
Asian	3.43	52
Mixed	71.14	1077
Others*	0.99	15
No classification	3.12	350
Year of death (%, n)		
2017	7.73	117
2018	17.17	260
2019	21.53	326
2020	24.90	377
2021	28.67	434
Season (%, n)		
Summer	27.13	411
Autumn	26.4	400
Spring	25.21	382
Winter	21.25	322
Education level (%, n)		
No education	2.84	43
Primary completed	6.27	95
Primary uncompleted	2.31	35
Secondary completed	21.8	330
Secondary uncompleted	4.16	63
Tertiary completed	17.31	262
Tertiary uncompleted	4.23	64
Not answered	41.08	622

Notes: *Others considered Amazonian, Aymara or quechua.

Table A4. Country of origin and location of fatalities among dead travellers in Peru (N=1,514) between 2017 and 2021

Variable	Percentage	Number of observations
Continent/country of origin (%, n)		
Africa	0.20	3
Angola	0.07	1
Ivory coast	0.07	1
Cabo Verde	0.07	1
Americas	78.99	1196
Venezuela	28.34	429
US	18.03	273
Chile	7.00	106
Argentina	5.88	89
Colombia	4.29	65
Ecuador	4.29	65
Asia	4.96	75
Japan	1.39	21
Indonesia	1.25	19
China	1.06	16
Europe	15.19	230
Spain	5.88	89
Italy	3.76	57
France	1.39	21
Oceania	0.66	10
Australia	0.59	9
New Zealand	0.07	1
Country of origin income level (%, n)		
HIC	44.06	667
LMIC	55.94	847
Province in Peru where travellers died (%, n)		
Amazonas	0.07	1
Ancash	2.78	42
Apurimac	0.07	1
Arequipa	2.85	43
Ayacucho	0.26	4
Cajamarca	0.60	9
Callao	6.29	95
Cusco	4.04	4.04
Huancavelica	0.07	1
Huánuco	0.13	2
ICA	2.05	31
Junín	1.19	18
La Libertad	5.50	83
Lambayeque	1.26	19
Lima	40.99	619
Loreto	1.85	28
Madre de Dios	0.53	8
Moquegua	0.60	9
Pasco	0.13	2
Piura	3.44	52
Puno	1.79	27
San Martin	0.53	8
Tacna	1.66	25

Tumbes	2.65	40
Ucayali	0.73	11
Not answered	17.95	271

Notes: HIC= High-income country. LMIC= Low- and middle-income country. n stand for number of observations. US= United States of America.

Figure A2. Total number of deaths over time adjusted to national incidence of travellers in Peru, by year

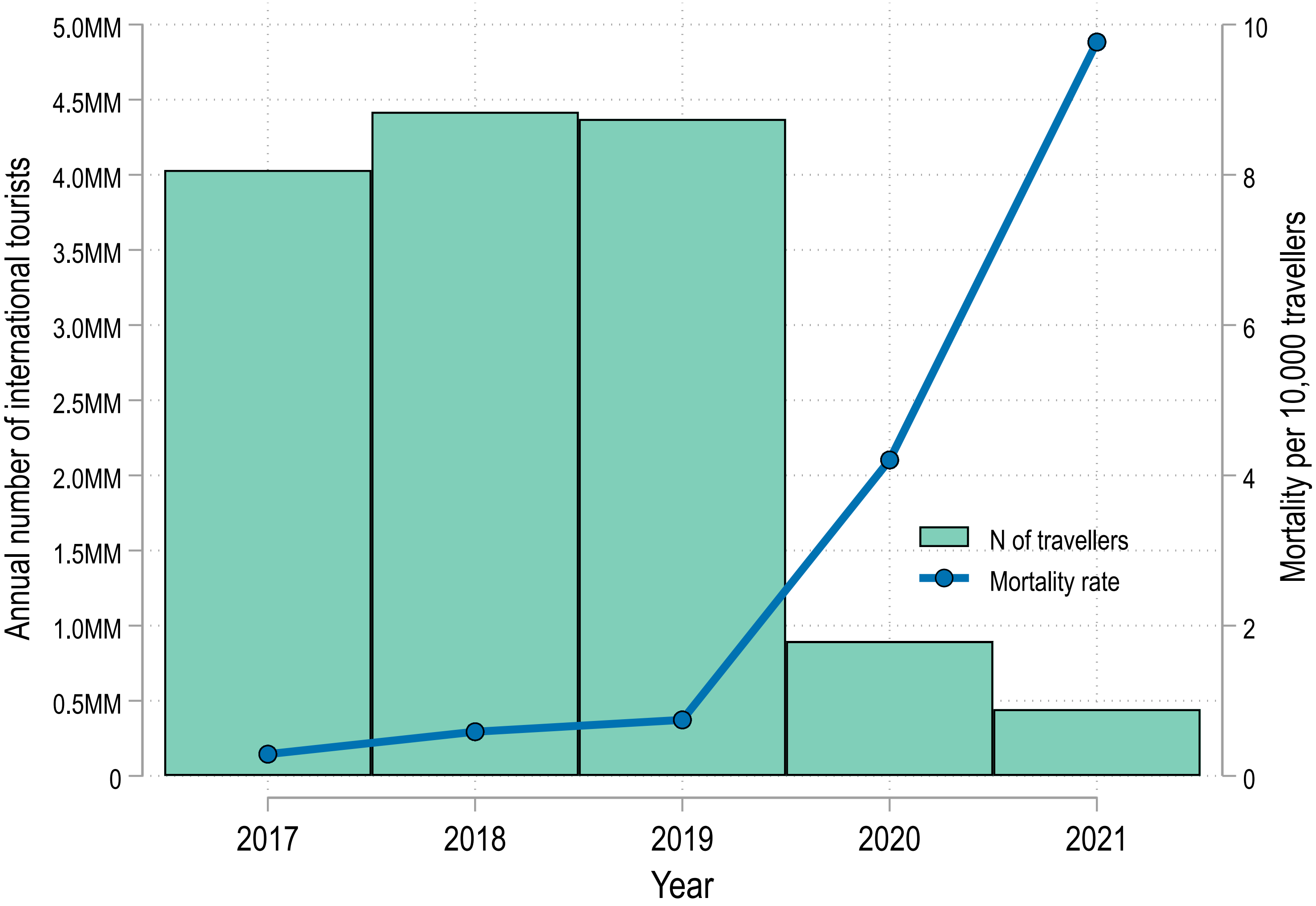

Notes: Total numbers of deaths included all causes of mortality (communicable, non-communicable, injuries, and unknown). MM= millions. Number of international tourists per year were extracted from Government data (2).

Figure A3. Number of deaths among travellers adjusted to total number of travellers in Peru between 2017 and 2021, by month.

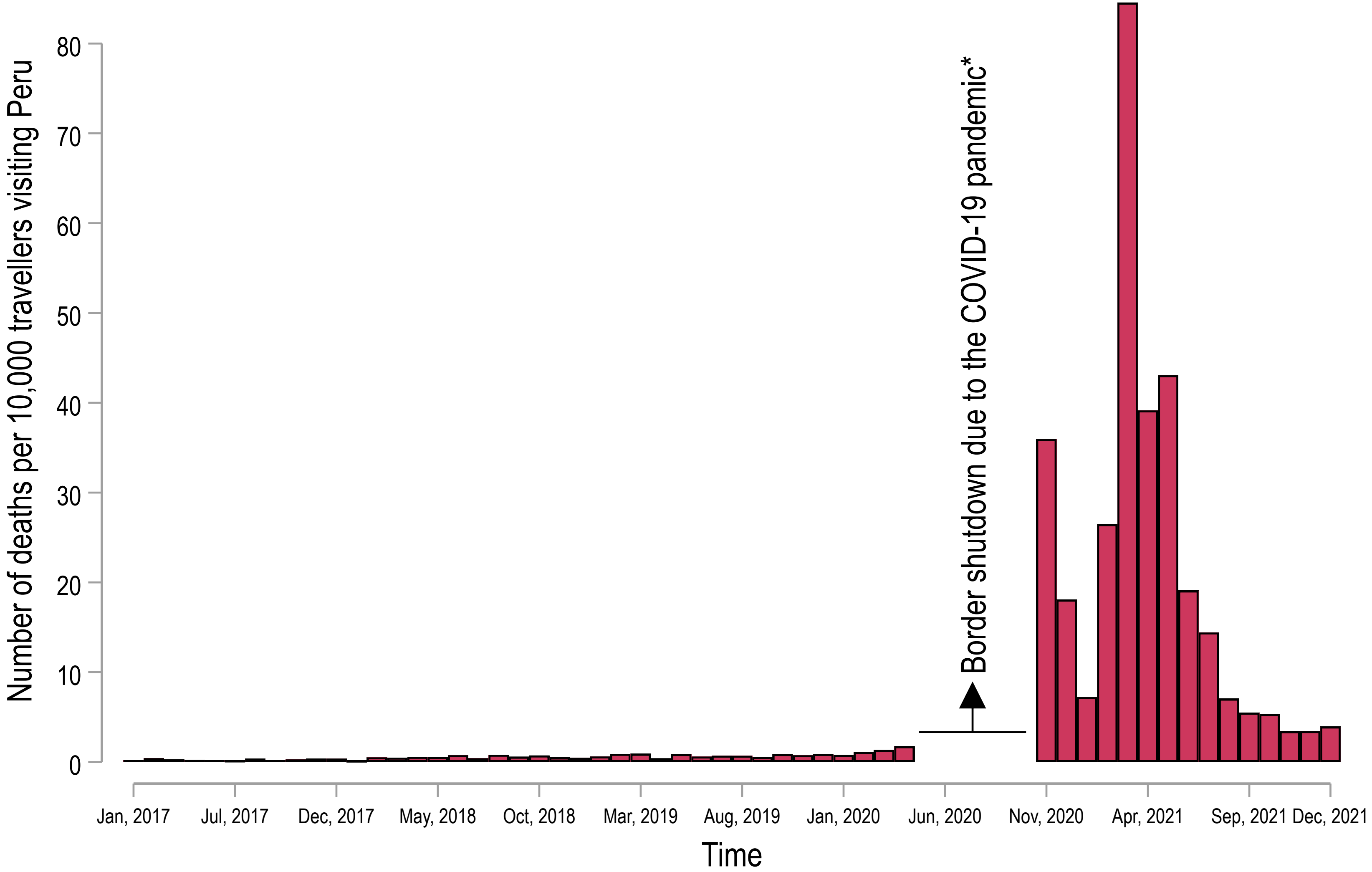

Notes: *There were no international tourists registered, during the COVID-19 pandemic. Number of tourists arriving in Peru extracted from the Ministry of foreign trade and tourism (2). Unadjusted monthly and annual change variation in number of deaths among travellers are shown in Figure A4.

Figure A4. Monthly and annual percentage variation in the number of total deaths, respect to the month or year from the reference year 2017

(A) Monthly percentage change compared to 2017's respective month

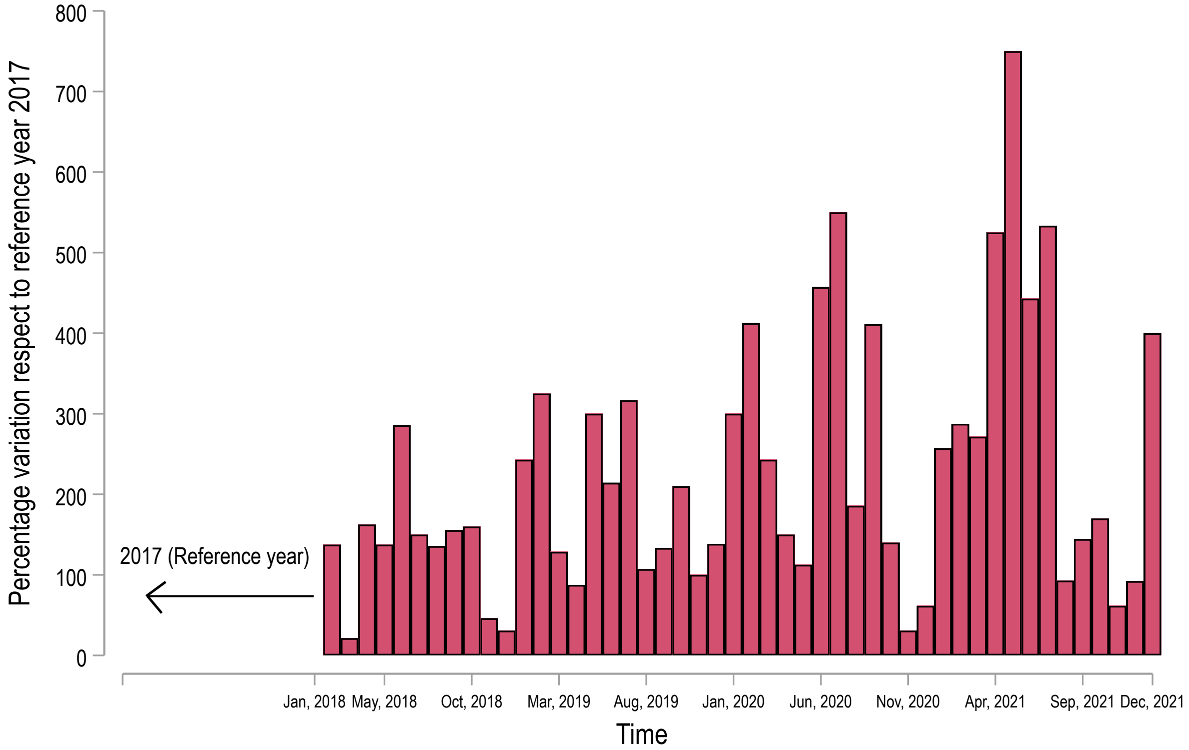

(B) Annual percentage change compared to 2017

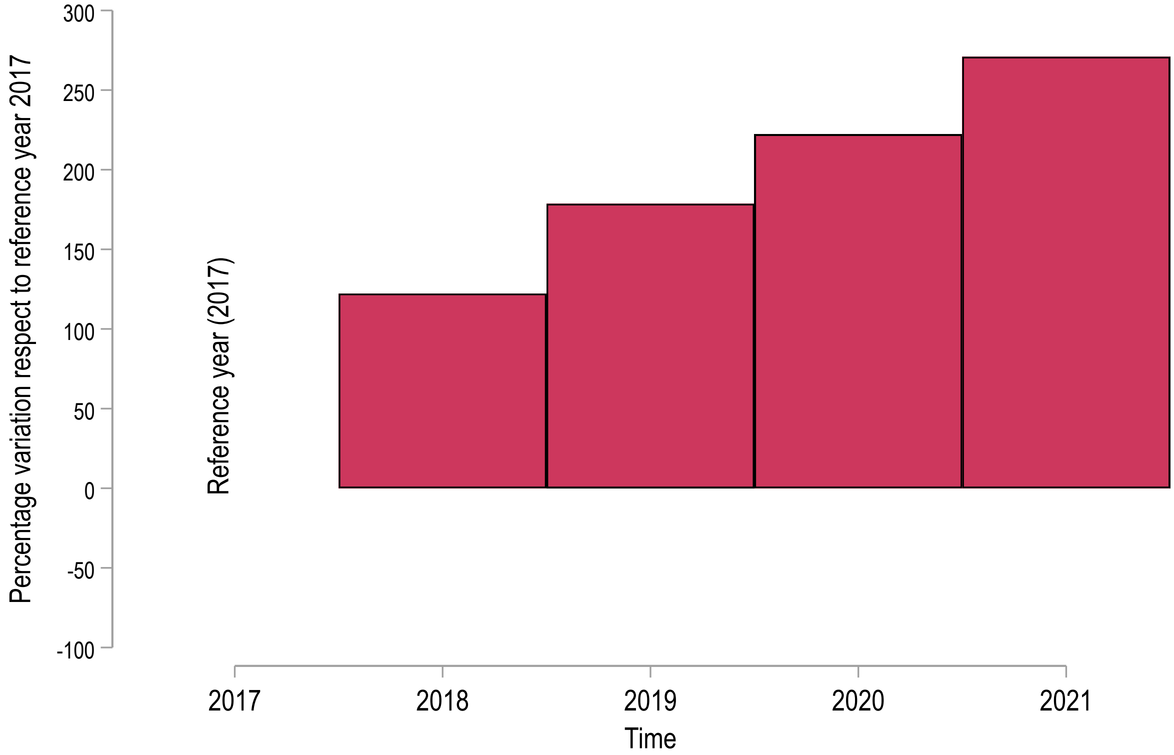

Notes: Monthly variations were computed respect to reference year 2017.

Figure A5. Number of deaths among travellers in Peru, by country origin

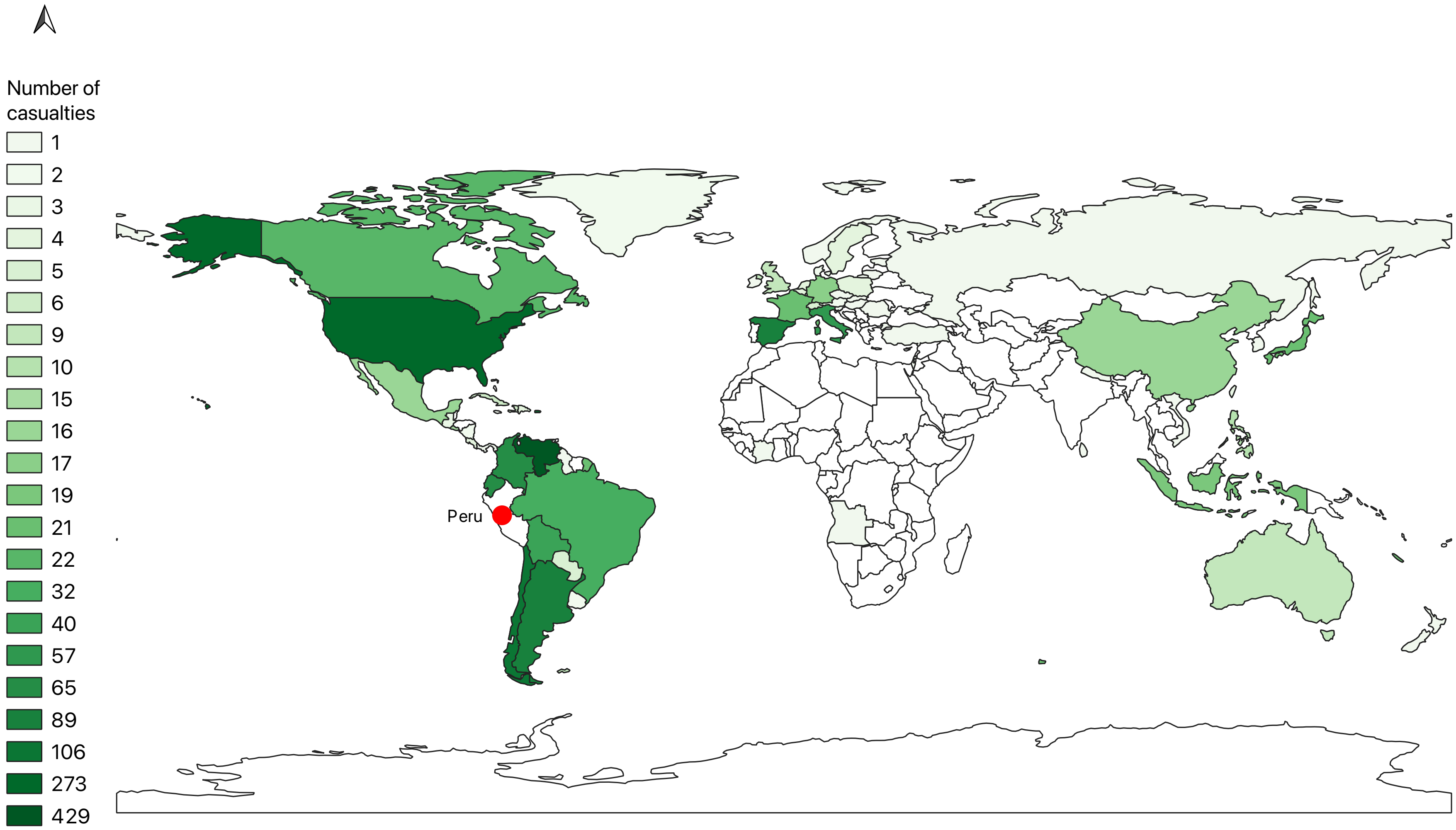

Notes: Darker colours indicate higher number of casualties per travellers' country origin.

Figure A7. Number of injuries-associated deaths among travellers adjusted to total number of travellers in Peru between 2017 and 2021, by month

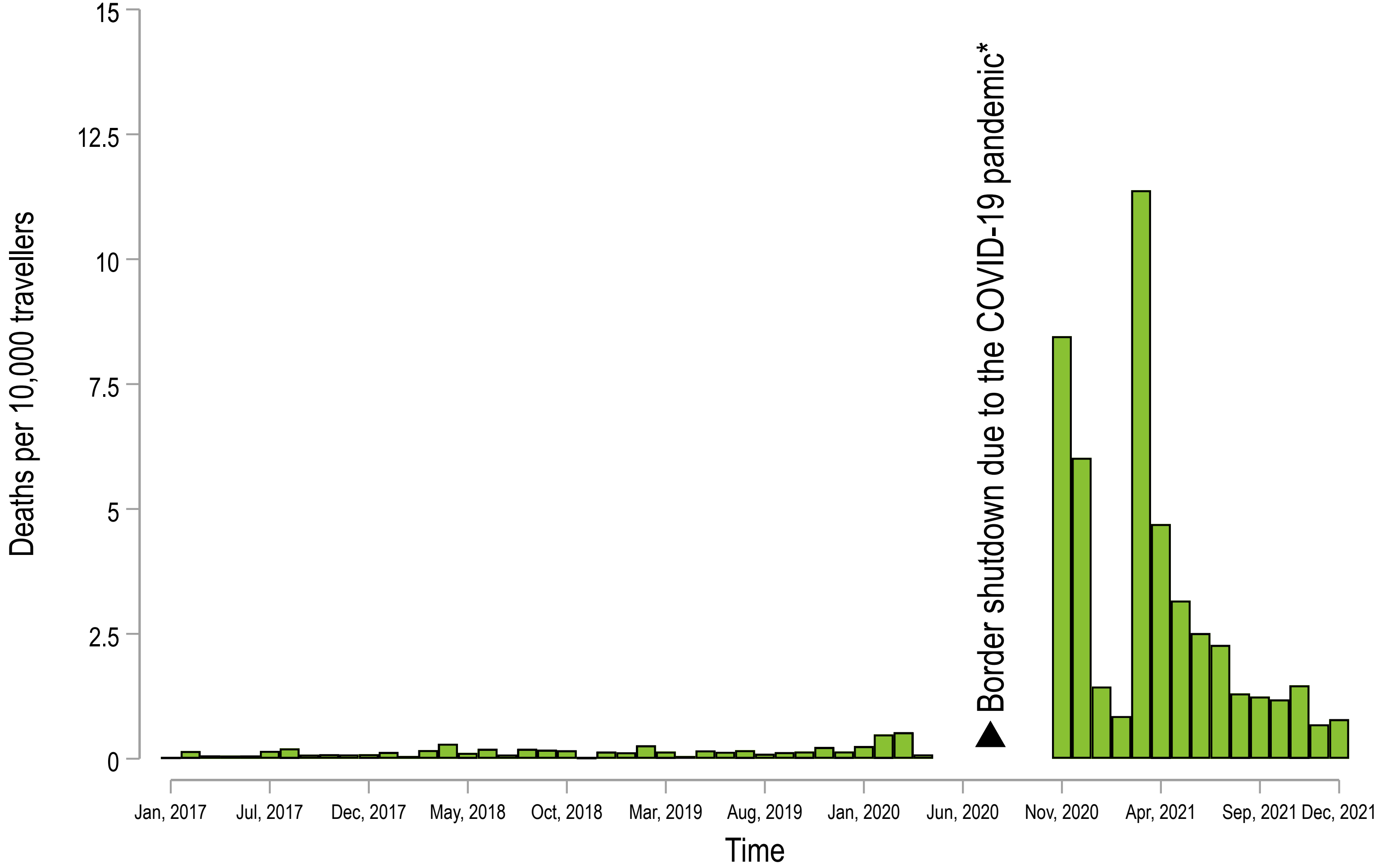

Notes: Data on annual number of international travellers were extracted from the Ministry of foreign trade and tourism (https://www.mincetur.gob.pe/centro_de_Informacion/mapa_interactivo/llegadaTuristasPais.html) Unadjusted monthly and annual number of deaths are shown in Figures A12 and A13 respect to the reference year 2017...

Figure A8. Number of non-communicable diseases-associated deaths among travellers adjusted to total number of travellers in Peru between 2017 and 2021, by month

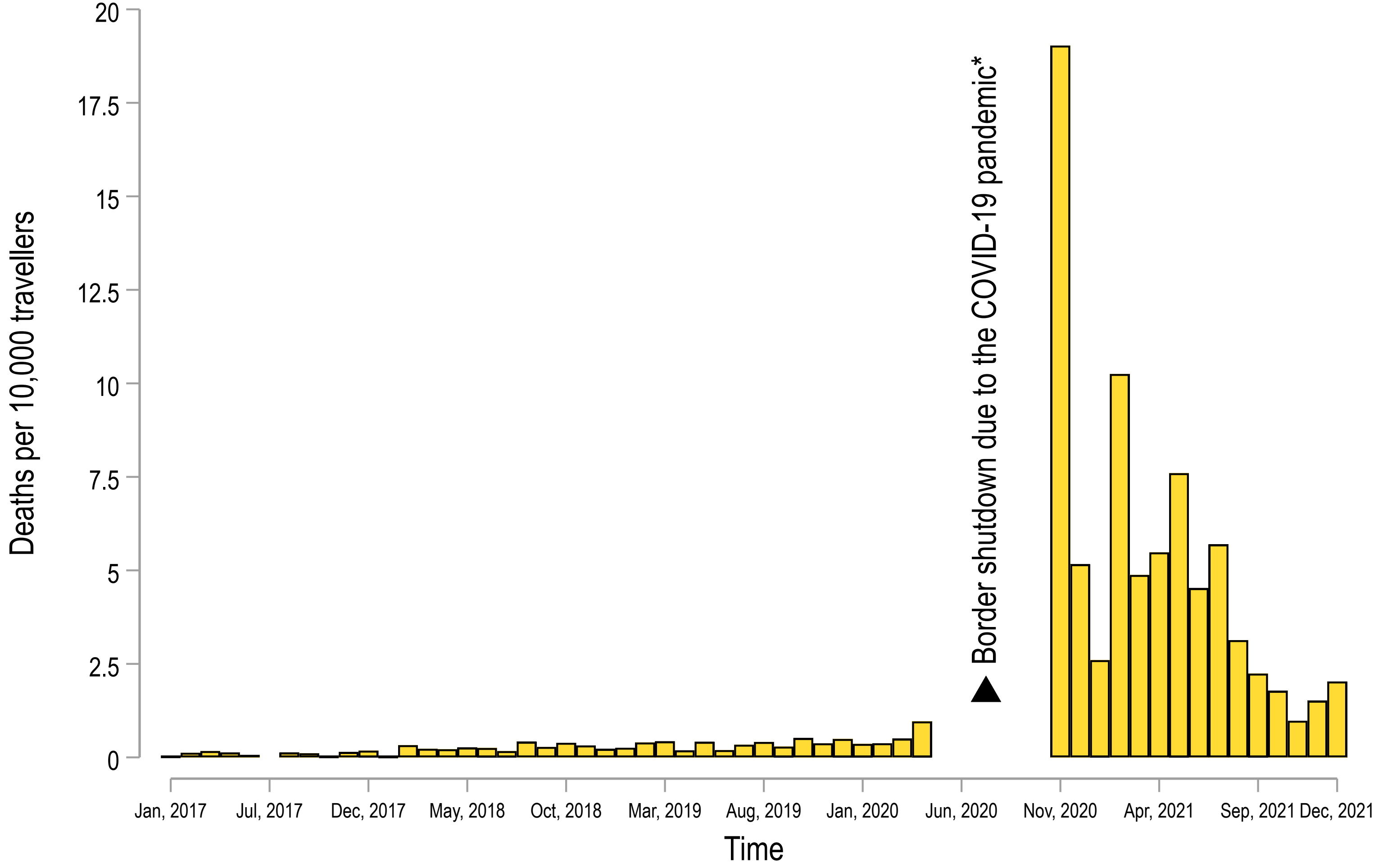

Notes: Data on annual number of international travellers were extracted from the Ministry of foreign trade and tourism (https://www.mincetur.gob.pe/centro_de_Informacion/mapa_interactivo/llegadaTuristasPais.html). Unadjusted monthly and annual number of deaths are shown in Figures A12 and A13 respect to the reference year 2017.

Figure A9. Monthly percentage variation in the number of total deaths, respect to the month from the reference year 2017, by death group

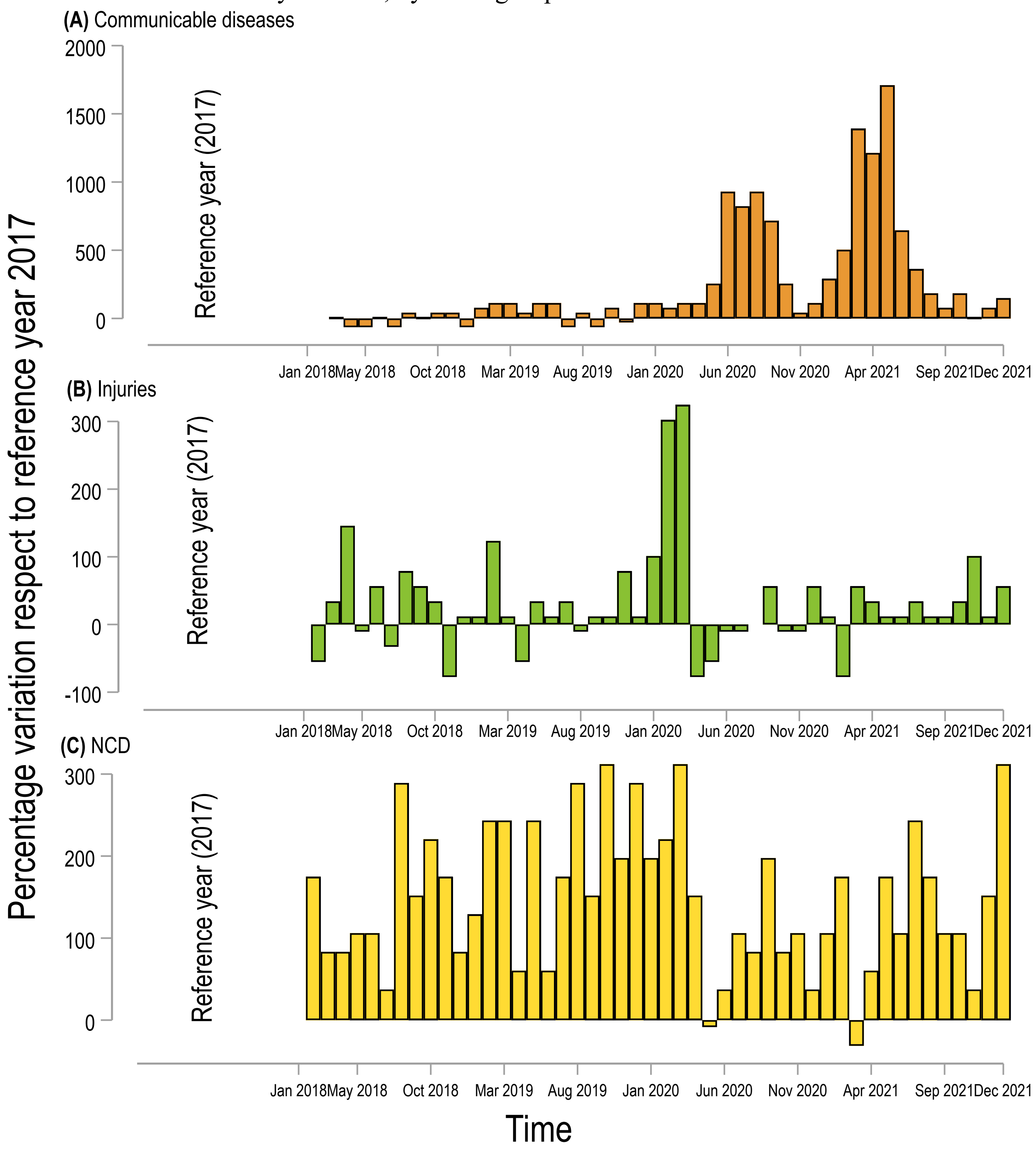

Notes: NCD= non-communicable diseases.

Figure A10. Annual percentage variation in the number of total deaths, respect to the reference year 2017, by death group

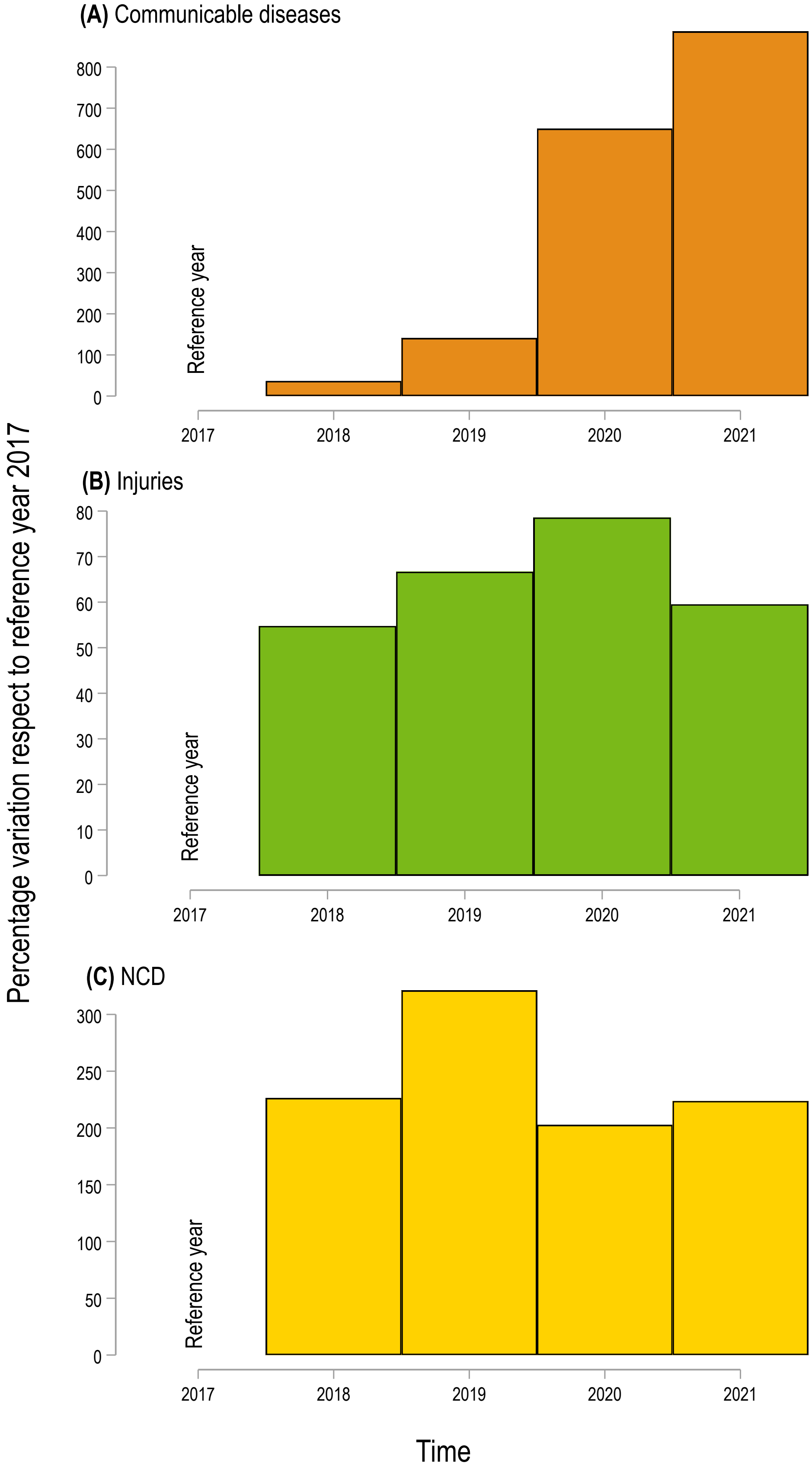

Notes: NCD= non-communicable diseases.

Figure A11. Causes of death among travellers accumulated overtime (2017-2021) excluding COVID-19-associated deaths in Peru, by death classification (N= 1, 366)†

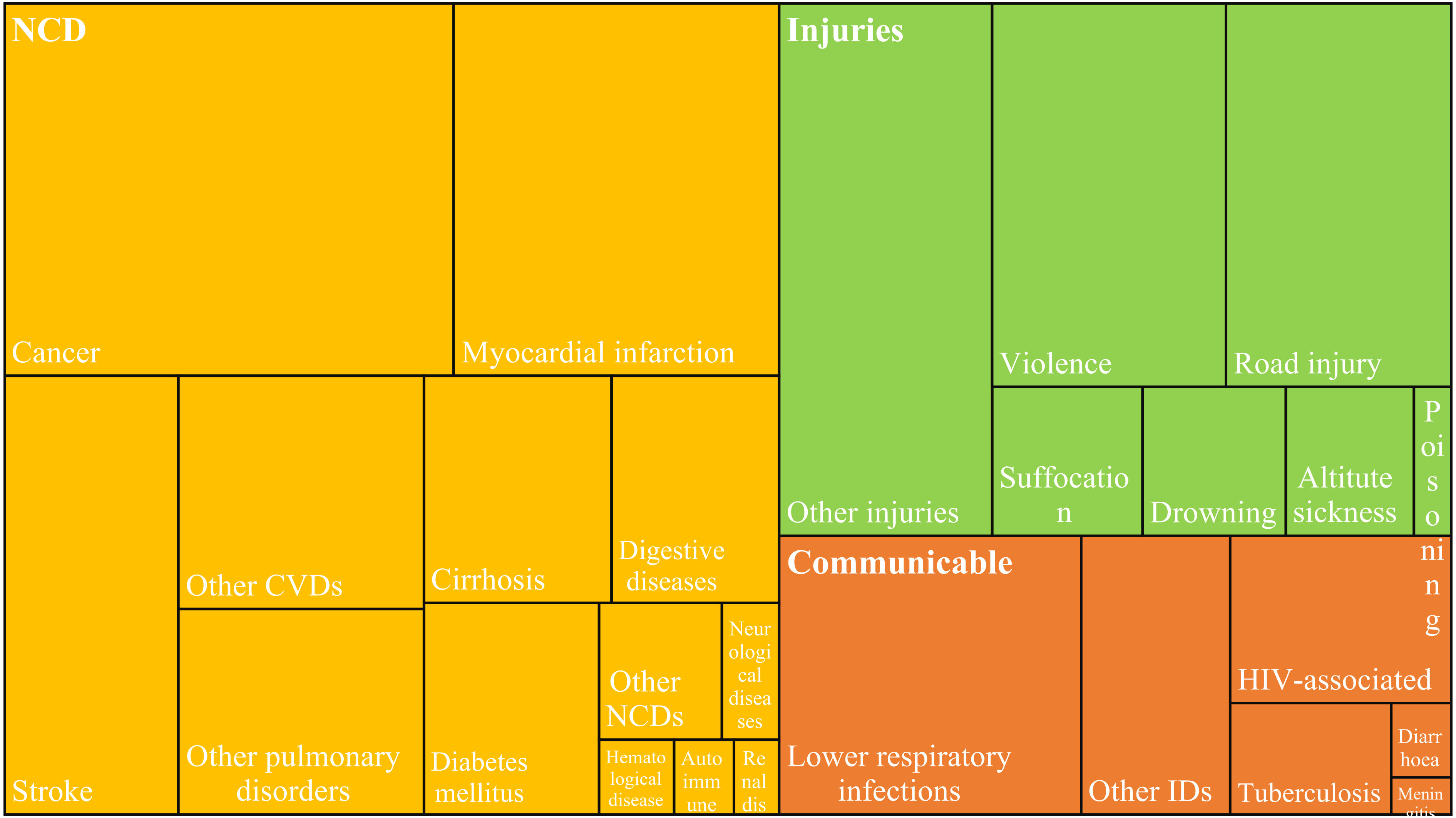

Notes: HIV= Human immunodeficiency virus, TB= Tuberculosis, CVD= cardiovascular disease, ID= Infectious diseases, NCD= non-communicable diseases. Counts per cause of death are shown under names. The onset of the COVID-19 pandemic was reported on the 6th of March 2020. †Deaths classified as "Others" were excluded from this analytical sample. Table 2 displays the exact numbers and proportions per death cause.

Figure A12. Distribution of the causes of death in Peru in both sexes and across all ages in 2019 according to the estimates from the Global Burden of Disease

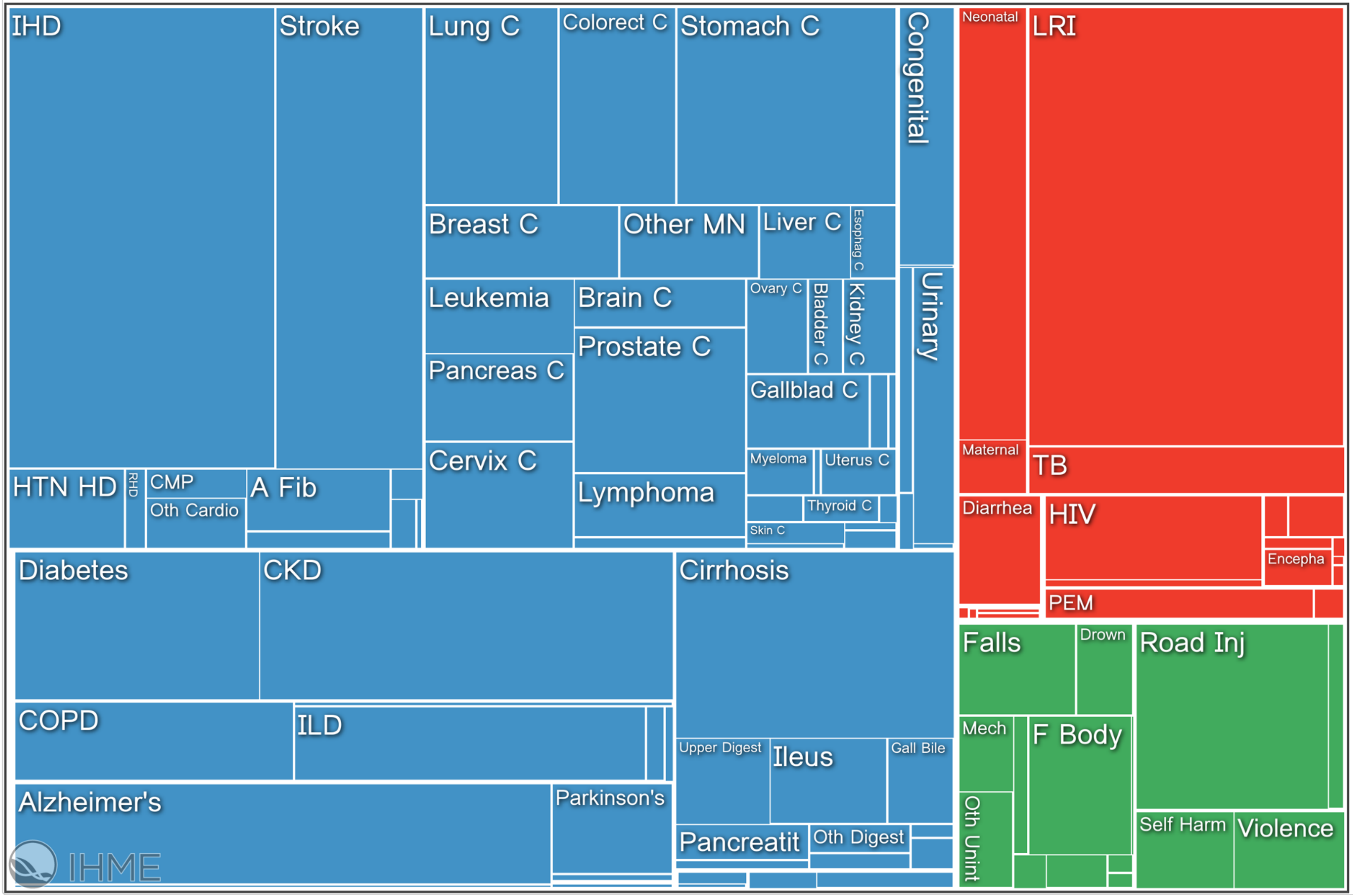

Notes: Blue stand for non-communicable diseases (NCDs), red is for communicable diseases and green for injuries. HIV= Human immunodeficiency virus, TB= Tuberculosis, CVD= cardiovascular disease, ID= Infectious diseases, NCD= non-communicable diseases. Counts per cause of death are shown under names. IHD= Ischaemic heart disease. COPD= Chronic obstructive pulmonary disorder. LRI= Lower respiratory infections. CKD= chronic kidney disease. ILD= Interstitial lung disease. HTN= Hypertensive heart disease. C= Cancer. MN= Malignant neoplasms. http://ihmeuw.org/66zp (3).

Figure A13. Main causes of death among travellers in Peru, by region and death classification using avoidable and non-avoidable mortality

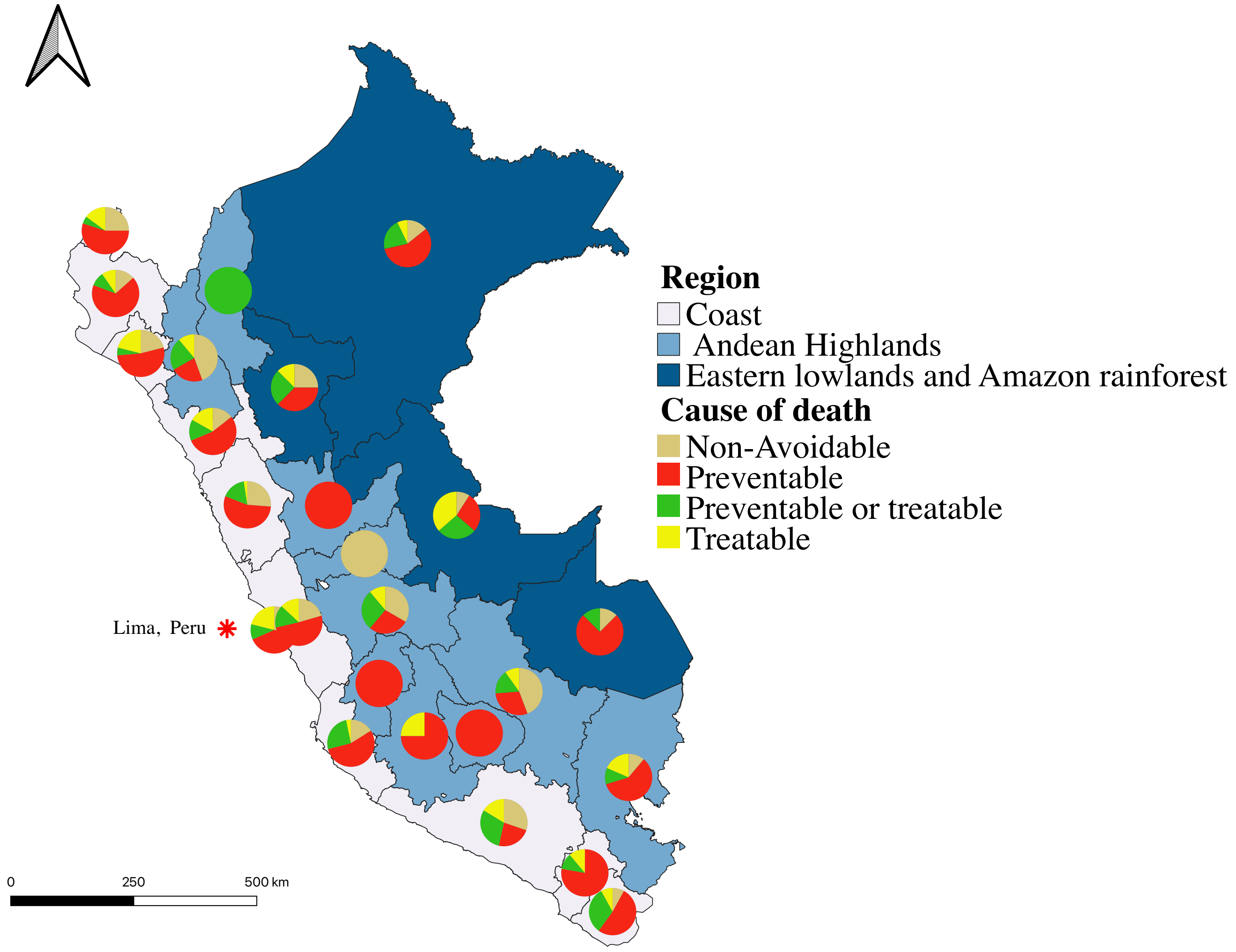

Notes: Preventable, preventable or treatable and treatable causes of death are considered avoidable. Causes of death were classified using the OECD/Eurostat lists of preventable and treatable causes of mortality (see Table A1 for more details.

Figure A14. Main causes of death among travellers in Peru over time, by death classification

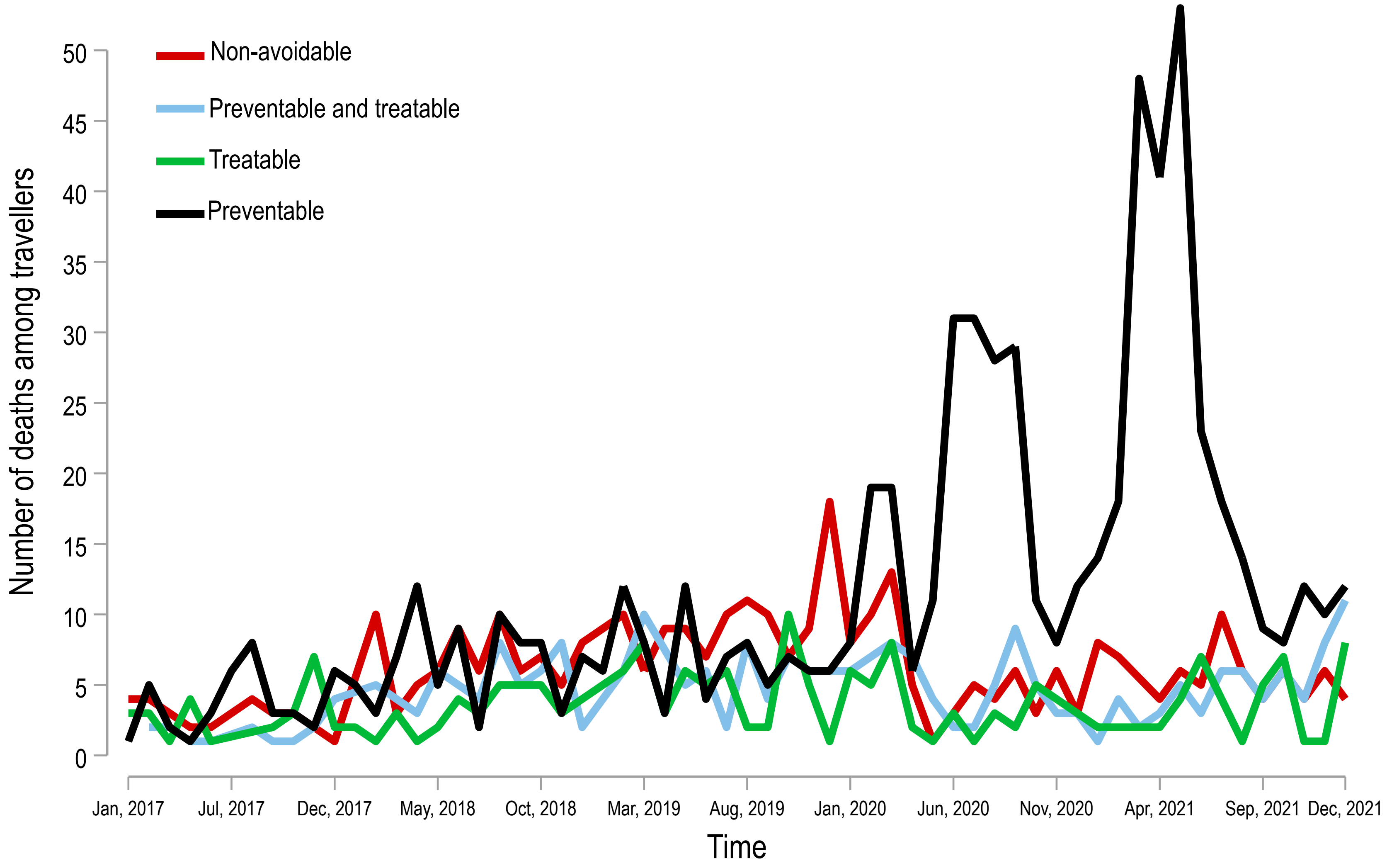

Notes: Causes of death were classified using the OECD/Eurostat lists of preventable and treatable causes of mortality (see Table A1 for more details).

Figure A15. Main causes of death among travellers in Peru over time, by death classification OECD!!!!

)

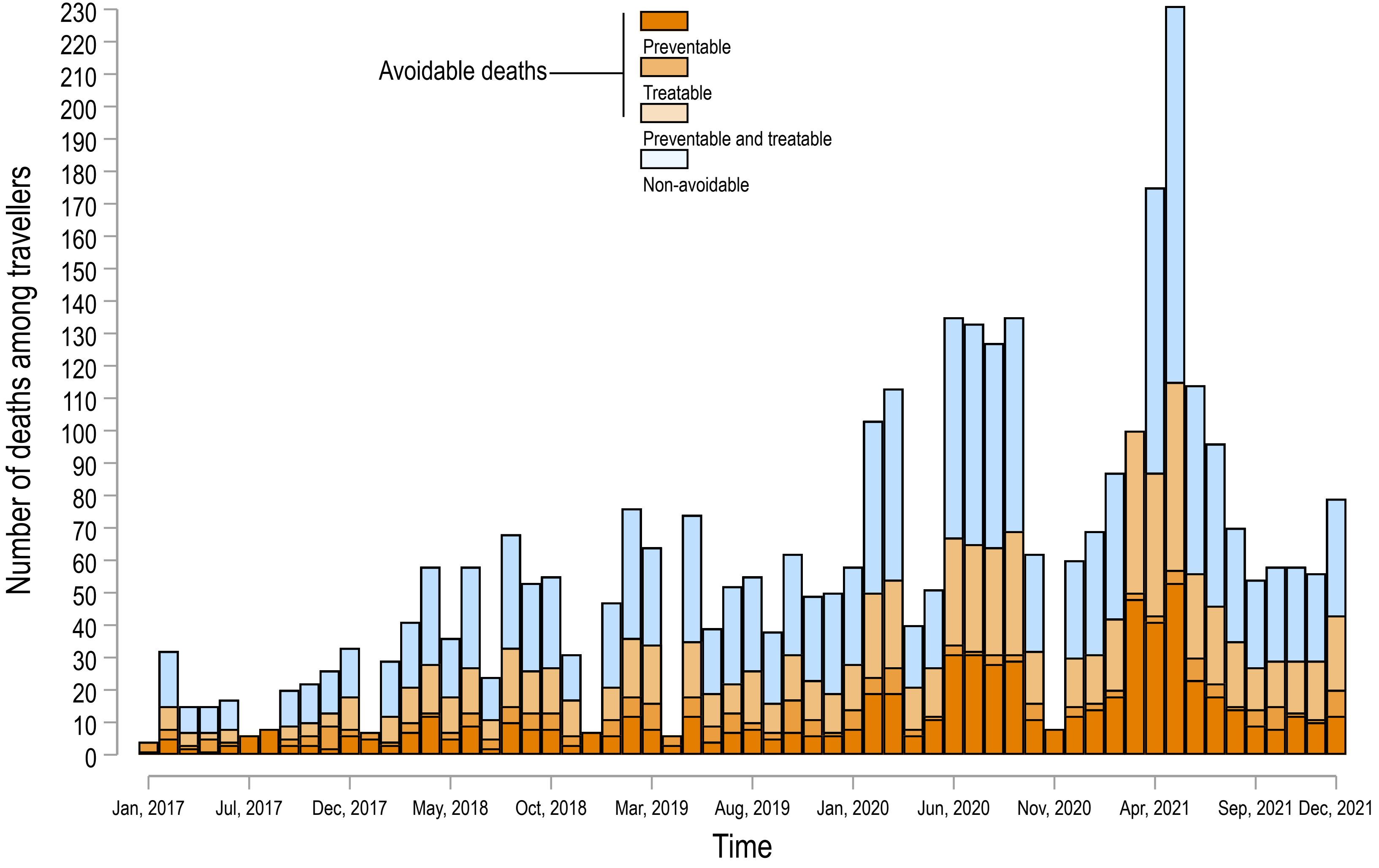

Notes: Avoidable deaths comprised preventable treatable and preventable or treatable.

Table A5. STROBE guidelines for observational studies

STROBE Statement—checklist of items that should be included in reports of observational studies (4).

	Item No.	Recommendation	Page No.	Relevant text from manuscript
**Title and abstract**	1	(*a*) Indicate the study's design with a commonly used term in the title or the abstract	1	All page
		(*b*) Provide in the abstract an informative and balanced summary of what was done and what was found	2	All page
**Introduction**			3	All page
Background/rationale	2	Explain the scientific background and rationale for the investigation being reported	3	First paragraph
Objectives	3	State specific objectives, including any prespecified hypotheses	3	Last paragraph
**Methods**			4	
Study design	4	Present key elements of study design early in the paper	4	First paragraph
Setting	5	Describe the setting, locations, and relevant dates, including periods of recruitment, exposure, follow-up, and data collection	4	First paragraph
Participants	6	(*a*) *Cohort study*—Give the eligibility criteria, and the sources and methods of selection of participants. Describe methods of follow-up<br>*Case-control study*—Give the eligibility criteria, and the sources and methods of case ascertainment and control selection. Give the rationale for the choice of cases and controls<br>*Cross-sectional study*—Give the eligibility criteria, and the sources and methods of selection of participants	4	First and second paragraph
		(*b*) *Cohort study*—For matched studies, give matching criteria and number of exposed and unexposed<br>*Case-control study*—For matched studies, give matching criteria and the number of controls per case	Not applicable	
Variables	7	Clearly define all outcomes, exposures, predictors, potential confounders, and effect modifiers. Give diagnostic criteria, if applicable	4	Second and third paragraph
Data sources/ measurement	8*	For each variable of interest, give sources of data and details of methods of assessment (measurement). Describe comparability of assessment methods if there is more than one group	4	First three paragraphs
Bias	9	Describe any efforts to address potential sources of bias	4	Statistical methods
Study size	10	Explain how the study size was arrived at	4	First paragraph

Quantitative variables	11	Explain how quantitative variables were handled in the analyses. If applicable, describe which groupings were chosen and why		
Statistical methods	12	(*a*) Describe all statistical methods, including those used to control for confounding	4-5	Statistical methods
		(*b*) Describe any methods used to examine subgroups and interactions	4-5	Statistical methods
		(*c*) Explain how missing data were addressed	4-5	Statistical methods
		(*d*) *Cohort study*—If applicable, explain how loss to follow-up was addressed *Case-control study*—If applicable, explain how matching of cases and controls was addressed*Cross-sectional study*—If applicable, describe analytical methods taking account of samplingstrategy	Not applicable	
		(*e*) Describe any sensitivity analyses		
**Results**				
Participants	13*	(a) Report numbers of individuals at each stage of study—eg numbers potentially eligible, examinedfor eligibility, confirmed eligible, included in the study, completing follow-up, and analysed	5	Results section, first paragraph
		(b) Give reasons for non-participation at each stage	5	First and second paragraph
		(c) Consider use of a flow diagram		Table 1
Descriptive data	14*	(a) Give characteristics of study participants (eg demographic, clinical, social) and information onexposures and potential confounders		Table 1, 3, Supplementary Tables A2-3
		(b) Indicate number of participants with missing data for each variable of interest	5	Table 1-2, Supplementary Tables A2-3
		(c) *Cohort study*—Summarise follow-up time (eg, average and total amount)		Figure 1 and Table 1-2
Outcome data	15*	*Cohort study*—Report numbers of outcome events or summary measures over time		
		Case-control study—Report numbers in each exposure category, or summary measures of exposure		
		Cross-sectional study—Report numbers of outcome events or summary measures	*5-6*	*Table 1-2, Supplementary Table A2-3*
Main results	16	(*a*) Give unadjusted estimates and, if applicable, confounder-adjusted estimates and their precision (eg, 95% confidence interval). Make clear which confounders were adjusted for and why they wereincluded	5-6	Table 1-2 and Supplementary materia
		(*b*) Report category boundaries when continuous variables were categorized	Not applicable	

		(*c*) If relevant, consider translating estimates of relative risk into absolute risk for a meaningful time period	Not applicable	

Other analyses	17	Report other analyses done—eg analyses of subgroups and interactions, and sensitivity analyses	Tables 1-2	Provided, by age-sex and sociodemographics.
**Discussion**				
Key results	18	Summarise key results with reference to study objectives	6-7	First fourth paragraphs in the discussion
Limitations	19	Discuss limitations of the study, taking into account sources of potential bias or imprecision. Discussboth direction and magnitude of any potential bias	8	Provided
Interpretati on	20	Give a cautious overall interpretation of results considering objectives, limitations, multiplicity of analyses, results from similar studies, and other relevant evidence	6-8	Provided
Generalisa bility	21	Discuss the generalisability (external validity) of the study results		
**Other information**				
Funding	22	Give the source of funding and the role of the funders for the present study and, if applicable, for the original study on which the present article is based	9	Provided

Notes: *Give information separately for cases and controls in case-control studies and, if applicable, for exposed and unexposed groups in cohort and cross-sectional studies.

Note: An Explanation and Elaboration article discusses each checklist item and gives methodological background and published examples of transparent reporting. The STROBE checklist is best used in conjunction with this article (freely available on the Web sites of PLoS Medicine at http://www.plosmedicine.org/, Annals of Internal Medicine at http://www.annals.org/, and Epidemiology at http://www.epidem.com/). Information on the STROBE Initiative is available at www.strobe-statement.org.

References

1. OECD/Eurostat commission. Avoidable mortality: OECD/Eurostat lists of preventable and treatable causes of death (January 2022 version). 2022.
2. Ministry of foreign trade and tourism Number of international tourists 2023 [Available from: https://www.mincetur.gob.pe/centro_de_Informacion/mapa_interactivo/llegadaTuristasPais.html.
3. Institute for Health Metrics and Evaluation (IHME). Global Burden of Disease Visualization tool GBD compare 2023 [Available from: http://vizhub.healthdata.org/gbd-compare.
4. Von Elm E, Altman DG, Egger M, Pocock SJ, Gøtzsche PC, Vandenbroucke JP. The Strengthening the Reporting of Observational Studies in Epidemiology (STROBE) statement: guidelines for reporting observational studies. The Lancet. 2007;370(9596):1453-7.
